# Fowl adenovirus (FAdV) fiber-based vaccine against inclusion body hepatitis (IBH) provides type-specific protection guided by humoral immunity and regulation of B and T cell response

**DOI:** 10.1186/s13567-020-00869-8

**Published:** 2020-12-02

**Authors:** Carlotta De Luca, Anna Schachner, Taniya Mitra, Sarah Heidl, Dieter Liebhart, Michael Hess

**Affiliations:** 1 Christian Doppler Laboratory for Innovative Poultry Vaccines (IPOV), University of Veterinary Medicine, Vienna, Austria; 2 Clinic for Poultry and Fish Medicine, Department for Farm Animals and Veterinary Public Health, University of Veterinary Medicine, Vienna, Austria

**Keywords:** Fowl adenovirus, fiber, inclusion body hepatitis, vaccine, humoral immunity, cellular immunity

## Abstract

A recombinant fowl adenovirus (FAdV) fiber protein, derived from a FAdV-8a strain, was tested for its efficacy to protect chickens against inclusion body hepatitis (IBH). FAdV-E field isolates belonging to both a homotypic (FAdV-8a) and heterotypic (-8b) serotype were used as challenge. Mechanisms underlying fiber-induced protective immunity were investigated by fiber-based ELISA, virus neutralization assays and flow cytometry of peripheral blood mononuclear cells, monitoring the temporal developments of humoral and cellular responses after vaccination and challenge exposure. Birds were clinically protected from the homologous challenge and showed a significant reduction of viral load in investigated target organs, whereas fiber-based immunity failed to counteract the heterologous serotype infection. These findings were supported in vitro by the strictly type-specific neutralizing activity of fiber immune sera. In protected birds, fiber vaccination prevented a post-challenge drop of peripheral B cells in blood. Furthermore, fiber immunization stimulated CD4^+^ T lymphocyte proliferation while moderating the CD8α^+^ T cell response and prevented challenge-induced changes in systemic monocytes/macrophages and γδ^+^ T cell subpopulations. Both vaccinated and adjuvant-only injected birds experienced a priming of systemic B cells and TCRγδ^+^ T lymphocytes, which masked possible pre-challenge effects due to the antigen. In conclusion, within FAdV-E, recombinant fiber represents a vaccine candidate to control the adverse effects of homotypic infection by eliciting an effective humoral immunity and regulating B and T cell response, whereas the failure of heterotypic protection suggests a primordial role of humoral immunity for this vaccine.

## Introduction

Fowl adenoviruses (FAdVs) are non-enveloped, dsDNA viruses belonging to the family *Adenoviridae*, genus *Aviadenovirus*. The current classification recognizes five species based on genomic criteria (*Fowl aviadenovirus A* to *Fowl aviadenovirus E* (FAdV-A–FAdV-E)), with 12 subordinate serotypes (FAdV-1 to -8a, and -8b to -11) defined by cross-neutralization [1, 2]. Particular FAdV types, belonging to different species, are associated with three disease complexes with relevance for the commercial poultry sector on a global scale [3]. Analogous to the extent of genetic separation between the responsible strains, adenoviral gizzard erosion (AGE) caused by FAdV-1 (species FAdV-A) represents a self-standing pathology, distinct from hepatitis-hydropericardium syndrome (HHS), caused by FAdV-4 (FAdV-C), and inclusion body hepatitis (IBH), caused by serotypes -2 and -11 (FAdV-D), and -8a and -8b (FAdV-E), which show relatively closer molecular relationship and similar features of pathogenesis and protection [3, 4].

To date, immunization strategies in the field are mainly limited to the use of inactivated autogenous vaccines, but the growing urgency for an efficacious and broad-coverage protection has led to the experimental development of FAdV subunit vaccines. Most of these efforts have utilized capsid components as target immunogens, based on the rationale that these are the main players in conferring antigenicity of adenoviruses [5]. In particular recombinant penton base and fiber, either alone or as a complex, were demonstrated to be efficacious vaccine candidates, providing the proof-of-concept with HHS as model system, with vaccination/challenge schemes based upon serotype -4 [6–8]. Subsequently, fiber-based vaccines were extended to IBH, addressing for the first time also vertical protection in the framework of a subunit vaccine [9]. However, differently from HHS with its mono-type etiology, the control of IBH by vaccination is complicated by a diverse spectrum of viral species and serotypes, ultimately requiring broad-protection strategies. The additional possibility of mixed infections in the field [10–12] indicates that chickens remain susceptible to heterologous infection despite pre-existing immunity against another FAdV serotype, and likely represents the molecular basis for the recently reported natural recombination of FAdVs, exchanging fibers between IBH-causing types [13]. Furthermore, enforced vaccination efforts against one serotype can cause a shift towards outbreaks with other serotypes [14–16]. On the other hand, experimental data on immunization against IBH collectively indicate a certain extent of heterotypic protection, albeit these studies remain ambiguous about coverage across the species boundary due to the use of bivalent FAdV-D/FAdV-E vaccines [17–19]. Cross protection amongst different IBH-causing serotypes was also reported using FAdV-C and FAdV-E strains as inactivated vaccines [20–22]. However, all these studies are based on whole virus as vaccine antigen, with protection likely resulting from a synergy of all antigenic components, an effect that does not apply for subunit vaccines.

Besides empirical demonstration of resistance to challenge of fiber-vaccinated birds, the immune mechanisms underlying protection are not well resolved. While both cellular and humoral immune responses are triggered by contact with live FAdV [23, 24], their participation in context with subunit antigens still needs to be clarified. Despite providing full protection, recombinant fiber derived from FAdV-4 elicited only moderate or no neutralizing antibodies in birds, raising questions about the immunological correlates of protection, especially outside the humoral repertoire, for such type of vaccines [7, 25]. Furthermore, type and number of fibers vary in a species-dependent manner, and this may prevent the extrapolation of results of recombinant fiber protection from the HHS to the IBH system.

The present study employed a recombinant fiber with genetic background of FAdV-8a to assess coverage against the complete, type-homologous (-8a) and -heterologous (-8b) spectrum of IBH. Moreover, this is the first study to extend the temporal profile of cellular and humoral immune responses to FAdV subunit vaccination beyond the time point of challenge, allowing the comparison between the pre-stimulated and the naïve (in our case, adjuvant-primed) response during infection.

## Materials and methods

### Virus and recombinant protein preparation

FAdV-E type reference strains TR59 (FAdV-8a) and 764 (-8b) (GenBank accession numbers KT862810 and KT862811) were used as template strains for cloning. Field isolates 11–16629 and 13–18153 (MK572859 and MK572862), identified by whole-genome sequencing and virus cross-neutralization as members of FAdV-8a and -8b, respectively [13], served as challenge strains. All strains were threefold plaque purified, propagated on primary chicken-embryo liver (CEL) cells, as described by Schat and Sellers [26], and viral titers were determined by endpoint titration [27]. Viral DNA for fiber gene cloning was extracted from cell culture supernatant with the DNeasy Blood & Tissue Kit (Qiagen, Hilden, Germany). The encoding regions for the FAdV-8a and FAdV-8b fiber (termed hereupon Fib-8a and Fib-8b) were cloned, expressed, purified and visualized as previously reported [7, 28].

### Animal experiment

Specific pathogen-free (SPF) broiler chicks were hatched from embryonated eggs (Animal Health Service, Deventer, The Netherlands) at our facilities and randomly divided into six groups (n = 18/group) as summarized in Table 1, separately housed in isolator units (HM2500, Montair, The Netherlands).

**Table 1:** Design of the animal experiment

Group	Designation	Vaccination	Challenge strain (serotype)
I	Vaccination-only	Fib-8a	–
II	Vaccine/homologous challenge	Fib-8a	11–16629 (FAdV-8a)
III	Vaccine/heterologous challenge	Fib-8a	13–18153 (FAdV-8b)
IV	Challenge control FAdV-8a	Adjuvant only	11–16629 (FAdV-8a)
V	Challenge control FAdV-8b	Adjuvant only	13–18153 (FAdV-8b)
VI	Negative control	–^a^	–

^a^ Not applicable.

At first day of life, chickens of groups I, II and III were vaccinated intramuscularly with 50 µg of recombinant Fib-8a protein, mixed 1:1 with GERBU Adjuvant P (GERBU Biotechnik GmbH, Heidelberg, Germany). Birds of groups IV and V (challenge controls) received a phosphate buffered saline (PBS)/adjuvant-mixture, and group VI (negative control) PBS instead. At 21 days of life (20 days post vaccination, dpv) birds were infected intramuscularly with 10^6.3^ 50% tissue culture infective dose (TCID_50_) of virulent FAdV-8a in groups II (vaccine/homologous challenge) and IV, and virulent FAdV-8b in groups III (vaccine/heterologous challenge) and V. Vaccination-only (group I) and negative control (group VI) were injected with PBS.

Five birds from each group were killed and necropsied at 3, 5 and 7 days post challenge (dpc), and the remaining birds on termination of the trial at 14 dpc. Birds that died due to infection were necropsied on the same day of their death. Endpoints for protection included clinical signs recorded during daily monitoring, *post mortem* findings, plasma analytes, and organ-body weight ratios for liver and spleen.

Coupled longitudinal monitoring of the humoral and cellular immune response in blood was restricted to the homologous protection setting, starting at 13 dpv with five vaccine recipients (from group I), five birds administered only adjuvant (group IV) and five non-vaccinated birds (group VI). At subsequent time points (20 dpv: prior to challenge, and 3, 5, 7, 14 dpc), serum for antibody investigation was collected from all the birds. Blood for investigation of cellular immunity was collected at the same time points in the homologous protection setting (groups I, II, IV, VI), keeping the sampled individuals consistent throughout the experiment (n = 5/group and all the remaining birds for the final measurement).

### Clinical chemistry

During killing and bleeding the birds, blood was collected from the jugular vein into heparin tubes (VACUETTE^®^, Greiner Bio-One, Kremsmünster, Austria). Plasma concentrations of aspartate transaminase (AST) and lipase were determined as previously described [29].

### Histopathology and immunohistochemistry (IHC)

Samples from liver, bursa of Fabricius and pancreas were fixed in 4% neutral buffered formalin and subsequently embedded in paraffin. In order to perform microscopic examination, 4–5 µm-thick tissue sections were cut with a microtome (Microm HM 360, Microm Laborgeräte GmbH, Walldorf, Germany) and mounted on glass slides before undergoing hematoxylin–eosin staining. Five birds from each group challenged with FAdV-8a (groups II and IV) and five birds from the negative control (group VI) were selected for histopathological analyses. All the selected birds were killed between 3 and 5 dpc, when the animals were most affected by the disease.

In order to identify virus-positive hepatocytes, liver sections were additionally mounted on coated glass slides (Superfrost ultra plus, Menzel Gläser, Braunschweig, Germany) to undergo IHC utilizing a polyclonal antibody against the FAdV-E/-7 reference strain YR36 (GenBank accession number KT862809) raised in rabbits, in a dilution of 1:5000. For this, the strain YR36 was propagated on primary CEL cells, pelleted through a CsCl cushion by ultracentrifugation, and the pellet dissolved in PBS. An equal amount of GERBU Adjuvant P (GERBU Biotechnik GmbH, Heidelberg, Germany) was added prior to repeated subcutaneous injections of rabbits. The paraffin was removed from the sections by sequential washing steps in ethanol 100%, 96%, 70%, and distilled water for 5 min each. The sections were then boiled for 10 min in citric acid-monohydrate buffer before being rinsed twice with PBS and left for 30 min in methanol + 1.5% H_2_O_2_, then 20 min in PBS. At that point the slides were covered in goat serum (Normal goat serum, Vector Laboratories, Burlingame, CA) and incubated in a humid chamber for 1 h; after removal of the serum, the sections were covered with the primary antibody solution and rested overnight in the humid chamber at + 4 °C. The following day, the tissue was washed in PBS and incubated for 30 min with the secondary antibody (Goat Anti-Rabbit IgG Antibody (H + L), Vector Laboratories, Burlingame, CA) and then 1 h with the ABC-reagent (ABC kit, Vectastain^®^, Vector Laboratories, Burlingame, CA) in the humid chamber before being washed in PBS. As a last step, the DAB substrate (DAB Substrate Kit, Vector Laboratories, Burlingame, CA) was applied and the reaction was monitored under the microscope before being stopped after 2.5 min by washing the slides in distilled water. A hematoxylin counterstaining was then executed for each slide.

### Quantitative polymerase chain reaction (qPCR) from tissues of target organs

Tissue samples from liver, spleen, pancreas and bursa of Fabricius were collected from birds challenged with FAdV-8a (groups II and IV) and from control birds (group VI) and stored at − 20 °C until processing. DNA was extracted with DNeasy Blood & Tissue Kit (Qiagen, Hilden, Germany) according to the manufacturer´s protocol and analyzed for quantification of viral DNA with a qPCR assay based on the 52K gene [30].

### Fiber-based enzyme-linked immunosorbent assay (ELISA) and virus neutralization test (VNT)

All sera collected during the trial were tested on recombinant fiber ELISA (coated with either Fib-8a or Fib-8b proteins, representing both types used for challenge) following the protocol described by Feichtner et al. [31].

Sera from all the vaccinated birds (groups I, II, III) immediately before challenge (20 dpv), and from 5, 7 and 14 dpc were also tested for neutralizing activity against the two reference strains (TR59 and 764) which served as a template for fiber expression, and the challenge strains (11–16629 and 13–18153) according to a protocol described earlier [7].

### Flow cytometry (FCM) analyses

Flow cytometry analyses on peripheral blood mononuclear cells (PBMCs) was performed on five birds per group within the homologous setting (group I, IV, VI at 13 dpv, and group I, II, IV, VI from 20 dpv onwards), keeping the sampled individuals consistent throughout the experiment.

#### Blood collection and preparation

For the separation of PBMCs, 2 ml of blood was collected from the wing vein of each bird in a heparin syringe. The blood was mixed with an equal volume of cold PBS, pH 7.4 (ThermoFisher Scientific, Vienna, Austria) with 2% fetal bovine serum (FBS) (ThermoFisher Scientific, Vienna, Austria). The prepared suspension was then slowly layered above a double volume of Histopaque^®^-1077 (Sigma-Aldrich, Vienna, Austria) for density gradient centrifugation. The cells from the interphase layer were collected and washed. Finally, the pellet was dissolved in 1 mL of the same solution.

#### FCM staining protocol

Mononuclear cells from the blood were examined for their viability using Nexcelom cellometer X2 fluorescent viability cell counter system (Nexcelom Bioscience, Manchester, UK). A concentration of 2 × 10^7^ cells/mL of PBS + 2% FBS was adjusted before the cells were stained. Different combinations of monoclonal antibodies (mAbs) were used for immunophenotyping of CD4^+^ T cells, CD8α^+^ T cells, B cells, monocytes/macrophages, TCRαβ^+^ T cells and TCRδγ^+^ T cells from the isolated cells. Gating strategy for PBMC is given as Additional file 1. A uniform gating hierarchy was used throughout all sampling days. Detailed information on antibody combinations and their fluorescence labelling by second-step reagents are given in Additional file 2. The final concentration of every antibody was determined by titration and the respective isotype controls were included.

For staining of mononuclear cells isolated from blood, 25 µl of the adjusted cell suspension was transferred into wells of 96-well microtiter plates (Sarstedt, Nümbrecht, Germany) together with the respective primary antibodies for incubation for 20 min at 4 °C. Afterwards, cell pellets obtained by centrifugation at 4 °C, 450×*g* for 4 min were washed two times with cold PBS + 2% FBS. For biotinylated antibodies, the secondary reagent Brilliant Violet 421™ Streptavidin (BioLegend, San Diego, CA, USA) was applied. Following another incubation step for 20 min at 4 °C, further washing was performed. The cells were fixed with BD fixation buffer (BD Biosciences, San Jose, CA, USA) according to the manufacturer’s protocol. Finally, the pellets were suspended in 200 µl cold PBS + 2% FBS kept at 4 °C until FCM analysis.

#### FCM analysis

FCM of stained cells was performed on a FACSCanto II (BD Biosciences, San Jose, CA) flow cytometer equipped by FACSDiva Software version 6.1.3 (BD Biosciences). At least 40,000 lymphocytes per sample were recorded. Analysis of FCM raw data was performed by FlowJo_V10 software (BD Biosciences, San Jose, CA). Absolute quantification of the cells was performed according to Mitra et al*.* [32].

### Statistical analyses

In order to verify the normal distribution assumptions, a preliminary analysis of the datasets was carried out using Shapiro–Wilk test associated with a visual inspection of histograms and normal Q–Q plots. The mean values from plasma analyses, liver- and spleen-body weight ratio, as well as cell populations in PBMC of vaccinated groups were compared with the negative control and their respective challenge control groups via unpaired Student’s *t*-test. Pairwise comparisons for datasets not meeting the normality assumptions were carried out with Mann–Whitney *U* test. In each case, *p* values ≤ 0.05 were considered statistically significant. Statistical analyses were performed with the software package SPSS Version 26 (IBM SPSS Statistics; IBM Corp., Armonk, New York, USA).

## Results

### Clinical protection of recombinant Fib-8a against homologous and heterologous challenge

Following challenge, clinical signs were characterized by mild depression in one of the birds from the vaccine/heterologous challenge group, one bird of the FAdV-8a and three birds of the -8b challenge controls between 4–5 dpc, and one bird that died at 3 dpc in the vaccine/homologous challenge group. No clinical signs were recorded in the vaccination-only and negative control group throughout the whole experiment.

Frequent gross pathological lesions included severe swelling, marble-like appearance and hemorrhagic areas in most of the livers from the infection-only groups, with a tendency of being more prominent at 3 dpc and less severe at 7 and 14 dpc. Necrotic foci were present on the liver of two birds from the challenge control -8a at 3 dpc and six birds from challenge control -8b between 3–7 dpc. Similar lesions were observed in the vaccinated/challenged groups as well, although the general affection of liver was milder and necrotic lesions were observed in only one bird of the vaccine/heterologous challenge group at 5 dpc. Pathognomonic lesions recorded in liver, spleen and pancreas are summarized in Table 2. No specific lesions were recorded in birds of the vaccination-only and negative control group at any time point.

**Table 2:** Summary of pathognomonic gross lesions recorded in necropsied birds at defined days post challenge (dpc)

	Liver dpc										Spleen dpc				Pancreas dpc				
	3			5			7			14	3	5	7		3	5	7		14
	Focal necroses	Hemorrhages	Marbled	Focal necroses	Hemorrhages	Marbled	Focal necroses	Hemorrhages	Marbled	Marbled	Marbled	Marbled	Marbled	Petechiae	Congestion	Hemorrhages	Congestion	Hemorrhages	Congestion
Vaccine/hom. chall.	–^a^	–	–	–	2/5	1/5	–	–	3/5	1/2	1/6	–	2/5	1/5	1/6	1/5	–	–	–
Chall. contr. -8a	2/5	2/5	2/5	–	–	4/5	–	–	3/5	1/3	–	–	2/5	–	–	–	–	–	–
Vaccine/het. chall.	–	–	3/5	1/5	1/5	1/5	–	–	5/5	-	–	–	1/5	–	–	–	–	1/5	1/3
Chall. contr. -8b	4/5	–	3/5	1/5	1/5	4/5	1/5	1/5	1/5	2/3	–	4/5	3/5	–	–	–	3/5	–	–
Neg. contr.	–	–	–	–	–	–	–	–	–	–	–	–	–	–	–	–	–	–	–

^a^ No lesions recorded.

**Figure 1:**
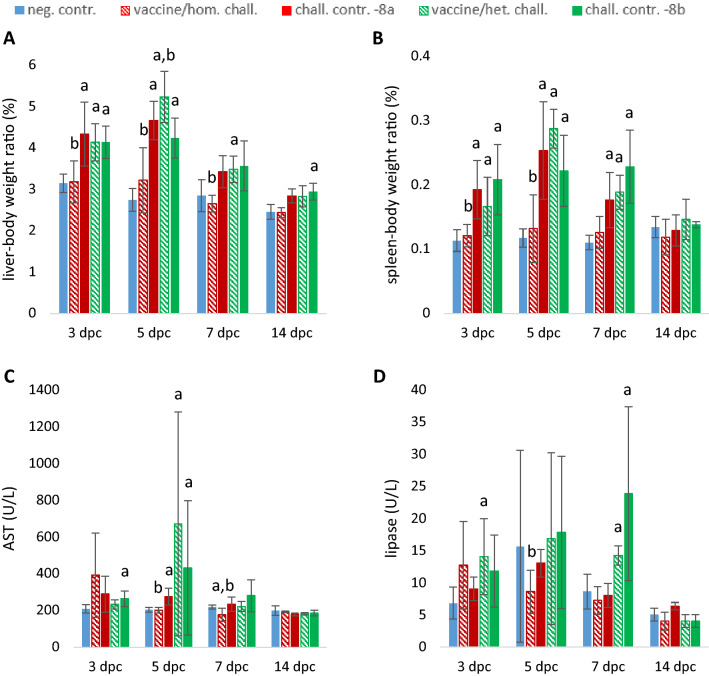
**Organs-body weight ratio and plasma analytes.** Mean and standard deviation of liver-body weight ratio (**A**), spleen-body weight ratio (**B**), AST in plasma (**C**) and lipase in plasma (**D**) measured for the six experimental groups at each time point. Significant differences against negative control are indicated with letter a, whereas significant differences against the respective challenge control are indicated with letter b.

Mean liver-body weight ratios were not affected in the vaccine/homologous challenge group, whereas they were significantly increased in all FAdV-8b infected groups and -8a challenge control at 3 and 5 dpc compared to the negative control (Figure 1). At 7 dpc only values from the vaccine/heterologous challenge group were still significantly increased. Similarly, spleen-body weight ratio was found elevated up to 7 dpc in all infected groups except the vaccine/homologous challenge. Plasma AST significantly increased at 5 dpc for the vaccine/heterologous challenge birds and the infection-only groups, whereas the lipase was mostly increased at 7 dpc for birds challenged with FAdV-8b.

### Viral load in target organs

**Figure 2:**
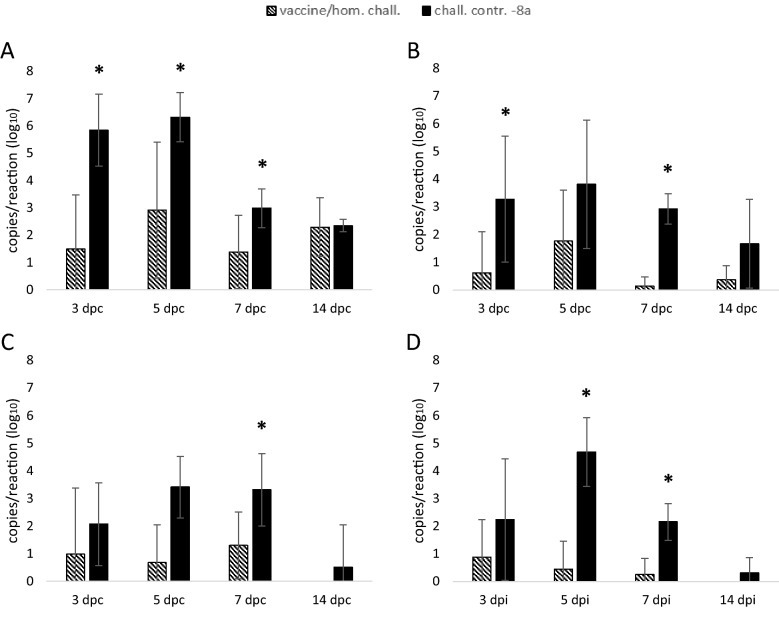
**Viral load in target organs.** Mean value and standard deviation of viral DNA expressed as copies/reaction (log_10_) measured in liver (**A**), spleen (**B**), pancreas (**C**) and bursa of Fabricious (**D**) for vaccine/homologous challenge group (II) and challenge control -8a (IV). Asterisks represent statistically significant differences.

The organ with the highest mean viral load was the liver at all time points post-challenge, except at 7 dpc, when the pancreas showed the highest viral load in the challenge control (Figure 2). However, in the vaccine/homologous challenge group the mean viral load in the liver was significantly reduced between 3–7 dpc compared to the challenge control, and a similar trend was recorded for spleen, pancreas and bursa of Fabricius, albeit not always statistically significant. At 7 dpc the viral DNA measured in the vaccinated birds was significantly lower (p ≤ 0.045) in all analyzed organs. No viral DNA was detected at any time point in the organs from negative control birds.

### Histopathology

Numerous areas with lymphocytic infiltration, and even necrosis in one case, were observed only in livers from challenge control FAdV-8a. Lymphocytic infiltration was also identified in the pancreas of only one out of five birds from the vaccine/homologous challenge group, in contrast with the challenge control where such lesions were noticed in four out of five birds, associated with necrotic areas in two cases. Necrosis was observed in the bursa of Fabricius of one challenge control bird, together with lymphoid depletion. Aggregation of viral material was observed via IHC in the nuclei of hepatocytes of all the five analyzed birds of the challenge control, whereas none of the tested vaccinated/challenged birds resulted positive. No lesions were recorded in the organs of the negative control. The microscopic lesions evaluated for each group are summarized in Table 3; histopathological changes in challenge control birds are exemplarily shown as Additional file 3.

**Table 3:** Summary of histopathological lesions observed in 5 birds/group euthanized between 3 and 5 dpc

	Liver			Pancreas		Bursa of Fabricius	
	Lymphoid infiltration	Necrosis	Virus-positive hepatocytes (IHC)	Lymphoid infiltration	Necrosis	Lymphoid depletion	Necrosis
Vaccin./hom. chall.	0/5	0/5	0/5	1/5	0/5	0/5	0/5
Chall. contr. -8a	5/5	1/5	5/5	4/5	2/5	1/5	1/5
Neg. contr.	0/5	0/5	x^a^	0/5	0/5	0/5	0/5

^a^ Not performed.

### Antibody development

#### Fiber-based ELISAs

**Figure 3:**
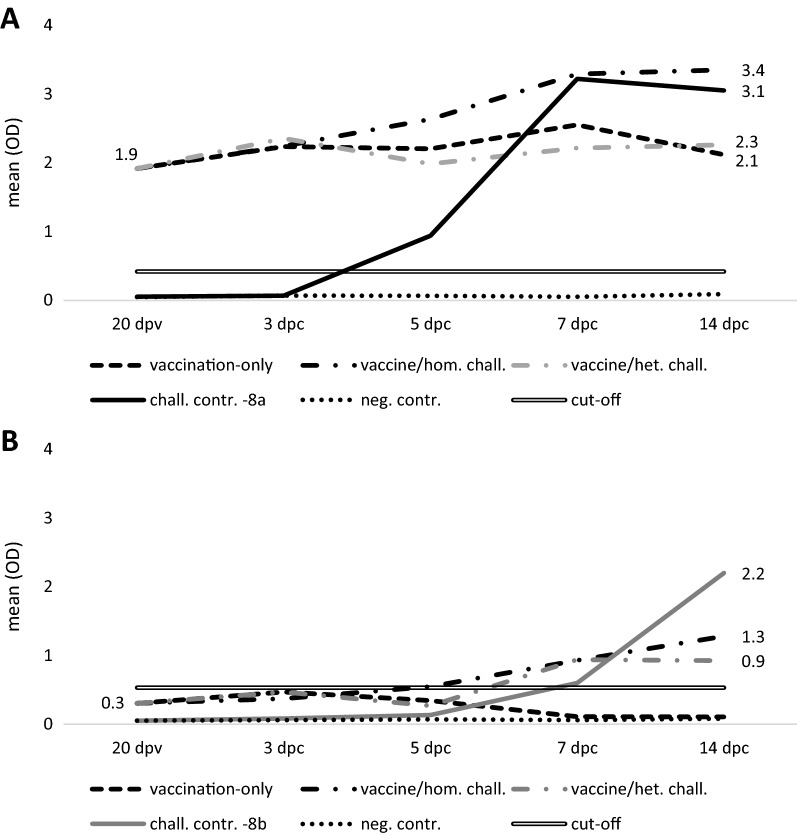
**ELISA-measured antibodies.** Results from ELISA coated with Fib-8a (**A**) and Fib-8b (**B**) respectively.

At the earliest measurement after vaccination (13 dpv), based on five birds from the vaccination-only group, one bird exhibited an OD above the cut-off defined earlier by Feichtner et al. [28] on the homologous ELISA (mean OD from five tested birds: 0.55 ± 0.62). However, at 20 dpv (immediately prior challenge) the mean OD of all vaccinated birds (groups I, II and III) was 1.92 ± 1.17, with the majority of birds (80%) being above the cut-off (Figure 3A). Notably, the only vaccinated bird that died after homologous challenge had no measurable antibodies. As expected, no antibody development was noted prior challenge in adjuvant-only administered birds (mean OD from birds of groups IV and V: 0.05 ± 0.01). Between 20 dpv and subsequent time points, mean Fib-8a ODs of the vaccination-only and vaccine/heterologous challenge group remained relatively constant, while the vaccine/homologous challenge group experienced a further rise throughout the post-challenge period up to OD 3.35 ± 0.02 at 14 dpc. In the FAdV-8a challenge control, a mean OD above cut-off was first noted at 5 dpc, eventually reaching a similar magnitude as the vaccine/homologous challenge birds.

A small subset of Fib-8a vaccinated birds (10%) exhibited cross-reactivity with the Fib-8b reactant, although with generally lower levels than the homologous reaction. Mean Fib-8b ODs of the FAdV-8b challenge control exceeded those of the vaccine/heterologous challenge group at 14 dpc (Figure 3B).

Negative control sera from all time points remained well below the earlier defined cut-offs.

#### VNT

**Figure 4:**
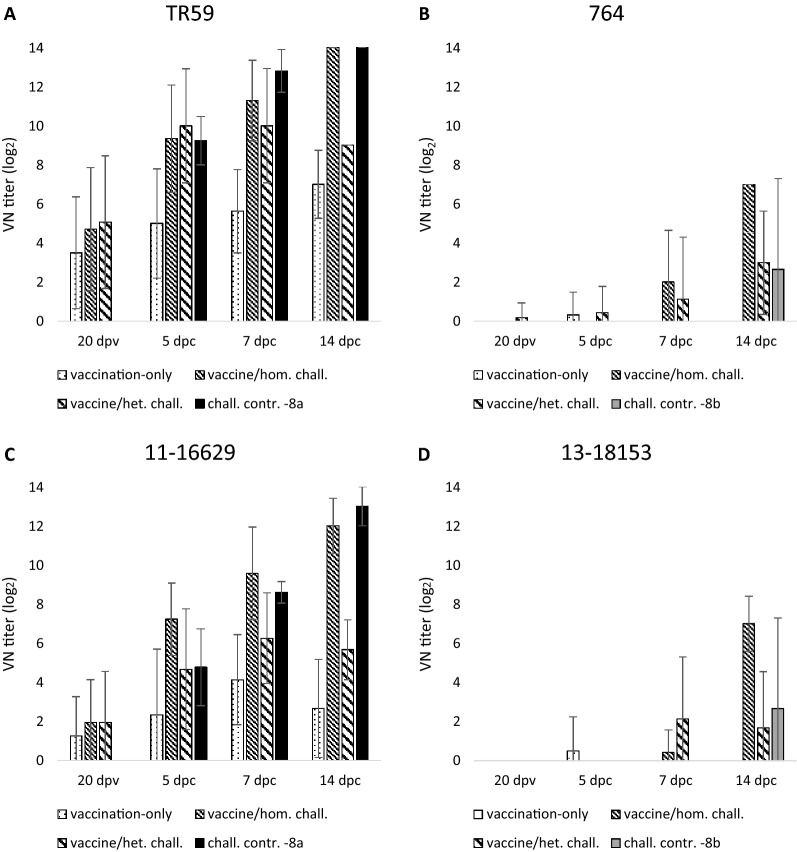
**Development of neutralizing antibodies.** Mean level and standard deviation of nAbs for each experimental group expressed in VN titers (log_2_) tested against different FAdV reference strains [TR59 (**A**), 764 (**B**)] and challenge viruses [11–16629 (**C**), 13–18153 (**D**)].

At 20 dpv, 73.5% of all vaccinated birds exhibited neutralizing antibodies (nAbs) against FAdV-8a/TR59 (mean titer 4.4 log_2_ ± 3.1) (Figure 4). A subset of these birds (38.8%) had nAbs against FAdV-8a/11–16629 (1.7 log_2_ ± 2.3), while only one vaccinated bird exhibited cross-neutralization with the lowest detectable titer level against FAdV-8b/764. In the vaccination-only group, mean nAb titers against TR59 continued to increase up to 7 log_2_ ± 1.7 until the end of the experiment, while mean nAbs against 11–16629 reached a maximum of 4.1 log_2_ ± 2.3 one week earlier and decreased afterwards. Upon challenge, 8a-specific nAbs of the vaccinated/FAdV-8a challenged group continuously increased, whereas titers of the vaccinated/FAdV-8b challenged group peaked at 7 dpc before decreasing, with generally lower levels compared to the homologous challenge. In the FAdV-8a challenge control, 8a-specific neutralizing activity was first recorded at 5 dpc and increased continuously until the end of the experiment, reaching values at the far end of the measured range (14 log_2_ ± 0 against TR59 and 13 log_2_ ± 1 against 11–16629).

Neutralizing activity against FAdV-8b was found in all vaccinated/infected groups independent of type of challenge. However, the vaccinated/FAdV-8a challenged group showed higher final titers than the vaccinated/FAdV-8b challenged birds (7 log_2_ ± 0 against 764 and 7 log_2_ ± 1.4 against 13–18153 vs. 3 log_2_ ± 2.6 and 1.7 log_2_ ± 2.9, respectively). In comparison, neutralizing antibodies were present in only one bird (8 log_2_ against 764 and 13–18153) of the FAdV-8b challenge control at 14 dpc.

### PBMC flow cytometry

#### B cells

**Figure 5:**
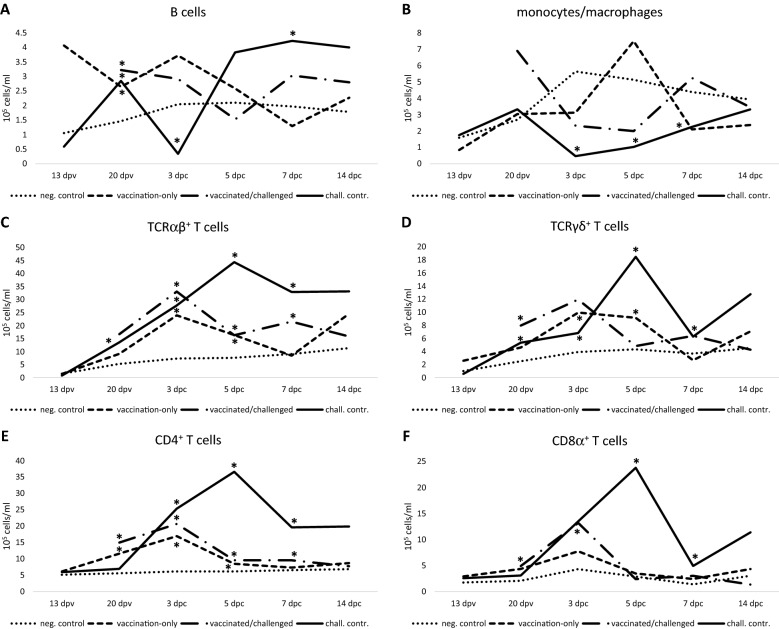
**Kinetics of cellular populations measured in PBMC throughout the study.** B cells (**A**), monocytes/macrophages (**B**), TCRαβ^+^ T cells (**C**), TCRγδ^+^ T cells (**D**), CD4^+^ T cells (**E**), CD8α^+^ T cells (**F**). Asterisks indicate significant differences compared to negative control group. No significant differences were recorded at 13 dpv and 14 dpc.

Means of the B cell population in blood were significantly higher at 20 dpv in all investigated groups (I, II, IV—homologous system) compared to negative control (basal level), with the values of vaccinated birds returning comparable to basal level from the subsequent time point (Figure 5A). After infection, a sharp and statistically significant decline was registered in the challenge control at 3 dpc, followed by a rapid increase, significant at 7 dpc. No significant changes were recorded at 13 dpv and 14 dpc. On an individual level, the highest intra-group deviation occurred at 13 dpv in vaccination-only birds, at 7 dpc for vaccinated/challenged birds, and at 5 dpc for challenge control (Additional file 4).

#### Monocytes/macrophages

Monocyte/macrophage populations in blood remained comparable between vaccinated groups and negative control throughout the whole experiment (Figure 5B). In contrast, the challenge control showed a decrease in the cell population following infection (from 3 to 7 dpc) and returned to basal level only at 14 dpc. Intra-group deviation was not prominent for challenge control birds, whereas in the vaccinated groups individual values showed a greater extent of variation (Additional file 5).

#### *TCRαβ*^+^*T cells*

TCRαβ^+^ T cells in PBMC significantly increased in the vaccination-only group at 23 and 25 dpv (corresponding to 3 and 5 dpc) before returning to basal levels (Figure 5C). The population was also consistently increased in the vaccinated/challenged group in the week following challenge. A similar tendency was recorded for challenge control birds, with a significant increase starting immediately before infection and peaking at 5 dpc, surpassing the vaccinated/challenged group, before returning to basal level at 14 dpc. No significant changes were recorded at 13 dpv and 14 dpc. Challenged birds tended to be subjected to higher individual variations (Additional file 6).

#### *TCRγδ*^+^*T cells*

Blood TCRγδ^+^ T cells significantly increased in vaccination-only birds at 23 and 25 dpv before returning to basal levels (Figure 5D). Immediately before challenge, the cell population increased in both subsequently infected groups. The vaccinated/challenged group did not show any other significant change, whereas levels of challenge control remained significantly elevated up to 7 dpc, peaking at 5 dpc before returning comparable to the negative control at 14 dpc. No significant changes were recorded at 13 dpv and 14 dpc. The highest intra-group variations were recorded at 5 dpc in the challenge control group (Additional file 7).

#### *CD4*^+^*T cells*

CD4^+^ T cells started to be significantly increased in PBMC at 20 dpv in both vaccinated groups before returning to basal levels at 27 dpv (corresponding to 7 dpc) and 14 dpc respectively (Figure 5E). Mean values of CD4^+^ T cells in challenge control significantly increased from 3 to 7 dpc and returned to basal level at 14 dpc. No significant changes were recorded at 13 dpv and 14 dpc. Intra-group deviation was also more prominent in the challenge control within the week after infection (Additional file 8).

#### *CD8α*^+^*T cells*

CD8α^+^ T cells of vaccination-only and negative control birds remained comparable at each investigated time point (Figure 5F). Birds of the vaccinated/challenged group showed a significant increase in their CD8α^+^ T cell population at 20 dpv and 3 dpc before returning to basal levels; challenge control birds showed a similar trend, with a sharp significant rise at 5 dpc, whereas values at 7 dpc were lower but still significantly increased compared to the negative control. No significant changes were recorded at 13 dpv and 14 dpc. Once again, challenge control birds tended to be subjected to higher individual variations (Additional file 9).

## Discussion

Inclusion body hepatitis (IBH) as a primary disease caused by certain FAdVs has become a growing concern to poultry industry in the past two decades, with an increasing documentation of outbreaks worldwide [3]. At the same time, intensified sequencing efforts, with mounting genomic data for FAdVs, have contributed to a more refined understanding of the diversity of strains involved in IBH. Based on their molecular composition, the spectrum of IBH strains encompasses types -2/-11 (FAdV-D), which constitute a narrow antigenic category due to being closely related [13], and the genetically much more divergent types -8a and -8b (FAdV-E). Given the greater antigenic variability of causative strains, the application of subunit vaccines, which are reasonably efficacious against HHS in experimental settings, should undergo a re-evaluation with regard to IBH. This is particularly relevant in case of the fiber, which was reported as the most type specific of all antigenic domains in FAdVs [13]. Furthermore, recombinant strains with exchanges in antigenic domains mark a possible gap in the established typing practices, which can lead to serious distortions when addressing cross-protection [13]. In light of this recent acknowledgment of recombinant FAdVs, all strains applied in this study were fully characterized with special focus on the relationship between fiber genes of vaccine and challenge strains.

Based on the serotype duality (FAdV-8a/-8b) of IBH, the present study shows that fiber-mediated response efficiently interferes with the homologous serotype infection even if it is a more distantly related strain of the same serotype, as shown by the preserved target organ-body weight ratios and levels of plasma analytes. At the same time, recombinant fiber fails to provide heterotypic coverage. Overall, vaccination could not completely prevent gross lesions in the target organs. Major lesions on the liver of vaccine/homologous challenge birds only appeared from 5 dpc, marking a delay in the onset of gross hepatic damage compared to the respective challenge control group. In contrast, vaccine/heterologous challenge birds presented hepatic lesions at each time point of the first week post infection.

Underlining the importance of antibodies and their type specificity in combating FAdV infection, the observed clinical protection in the homologous system (or, vice versa, its absence in the heterologous system) correlated well with in vitro findings. Although neutralizing titers of up to 10 log_2_ were found in vaccinated birds, they generally failed to cross-neutralize FAdV-8b. Despite potent homologous neutralization (up to 14 log_2_) from 5 dpc onwards, which possibly led to the significant decrease of viral load in target organs of vaccinated birds infected with homologous challenge, cross-reactivity was not evident before 7–14 dpc, but then also occurred in some sera with intermediate homologous titers. This indicates that immune sera containing only fiber antibody fractions, even if potently neutralizing, remain type-specific and thus less favorable to induce cross-protection, although this should be confirmed for other antigenic settings.

Efficacious fiber-based vaccines have been frequently associated with seroconversion in ELISA, but not necessarily neutralizing activity, as shown for the HHS/FAdV-4 system using fiber-2 as vaccine antigen [7, 25, 33]. Contrarily, induction and vertical transfer of nAbs, conforming with progeny protection, was reported for a prime-boost regimen with FAdV-8b fiber [9], and fiber-based neutralization is achieved independent of a second contact with the antigen, as our results show. However, variations in number and types of FAdV fibers suggest that controversial findings are due to differences in individual fibers´ immune functions, depending on the usage of a system with two types (FAdV-4) or one type of fiber (FAdV-8a/8b).

Data on PBMC cellular immune subpopulations stimulated by FAdV fiber subunits are so far limited to reports on a proliferation in CD4^+^, besides unchanged CD8α^+^, T lymphocytes following vaccination with FAdV-4 fiber-2 [25], and an increase in CD8α^+^ T cells after booster immunization with FAdV-8b fiber [9]. However, these findings only refer to vaccinated but non-infected birds, and do not account for the role of the adjuvant, which can have a self-standing effect on the chicken immune system [34–36]. The importance of the adjuvant is illustrated by the present study, which shows that, except for a pre-challenge increase in CD4^+^ T cells obviously related to fiber-dependent priming, fiber-vaccinated birds were distinctive from adjuvant-only administered birds only after challenge. In our setting, immune stimulation of the adjuvant was evident by an increase in B cells and TCRγδ^+^ T cells compared to the negative control. However, upon challenge, the non-specific nature of this stimulation was exposed, with challenge control birds experiencing an acute and significant drop in B cells and monocytes/macrophages compared to the negative control. This could be explained by the immunosuppressive effect of FAdVs [24, 37–39] and/or recruitment of immune cells to the target organs. The decrease of peripheral B cells was followed by a rise well above the level of the vaccinated/challenged group, in which B cells remained unchanged vs. the vaccination-only group and comparable to the negative control, while maintaining a continued production of antibodies. Final peaks of the antibodies were comparable between protected birds and their challenge control, however, the actual levels are likely masked by reaching saturation of the ELISA. This makes the post-challenge antibody development not a conclusive marker for protection in the applied setting. However, the steep rise of systemic B cell levels in the challenge control birds, immediately upon recovery within the week following challenge, highlights the importance of humoral effectors for limiting the infection in response to replicating virus.

Similar to B cells, a significant post-challenge drop in blood monocytes/macrophages, lasting up to 7 dpc, was prevented by the vaccine, with both vaccinated groups remaining comparable to the negative control. TCRαβ^+^ and TCRγδ^+^ T cells were vigorously stimulated early after viral challenge, and to a lesser extent by the vaccination. This was observed mainly in the vaccination-only group at 23 and 25 dpv (corresponding to 3 and 5 dpc), indicating that certain vaccine-induced effects may have overlapped closely with the time point of challenge. However, in the absence of an adjuvant-only control beyond 20 dpv, it cannot be confirmed that stimulation of TCRαβ^+^ and TCRγδ^+^ T cells was exclusively antigen-specific and their role in protection remains more speculative. Similarly, a CD4^+^ T cell proliferation was detected in response to both challenge and vaccination, with the difference that T helper cells were already distinctive for vaccinated groups at 20 dpv, and could thus serve as an indicator for subsequent protection.

CD8α^+^ T cell proliferation due to vaccination was noted, but only in one of the groups. In another study this effect was obtained only after booster [9], suggesting the necessity of robust sequential fiber administration for priming of CD8α^+^ T cells to activate cytotoxic T lymphocytes as a defense mechanism. Non-vaccinated birds relied on a vigorous cytotoxic defense, at least at a systemic level, reflected by the abrupt, steep rise of CD8α^+^ T cells in the challenge control group, whereas lower levels were reached in vaccinated/challenged birds before returning to basal level.

In conclusion, our data suggest that resistance to infection conferred by fiber critically depends on a humoral response by type-specific virus neutralization, which can also be linked to systemic B cell and CD4^+^ T cell priming by vaccination. On the individual birds’ level, this hypothesis is also supported by the death of a bird that had no vaccine-induced antibodies prior to challenge. The faster recruitment of humoral effectors in vaccinated birds, with more rapid clearance of virus, may contribute to limiting cytotoxic responses and immune-mediated tissue damage during progressive infection. The reliance on humoral immunity with a specific antibody fraction, however, explains why fiber fails to protect across the serotype boundary, imposing certain obstacles to use fiber as vaccine candidate for broad protection against IBH.

## Supplementary information

**Additional file 1. Gating strategy for peripheral blood mononuclear cells in multicolor flow cytometry analysis applying three different panels of antibody combination.** The cells were gated according to their light scatter properties. Potential leukocytes were gated with FSC/SSC and afterwards for CD45^+^ cells (double lined). In the first panel, CD45^+^ cells were further analyzed for CD45^+^CD4^+^CD8α^−^ T cells (orange gate) and CD45^+^CD4^−^CD8α^+^ T cells (blue gate). In the second panel, B cells and monocytes/macrophages were identified by CD45^+^Bu1^+^Kul01^−^ (purple gate) and CD45^+^Bu1^−^Kul01^+^ phenotype (green gate) respectively. The last panel analyzed CD45^+^TCRαβ^+^TCRδγ^−^ T cells (brown gate) and CD45^+^TCRαβ^−^TCRδγ^+^ T cells (pink gate). The gating strategy is shown as a representative example for isolated PBMCs from a bird at 14 dpc and was performed accordingly for all analyzed samples.

**Additional file 2. Antibody panels.** List of antibodies and antibody combinations used in this study.

**Additional file 3. Histopathological lesions in different organs from a challenge control bird infected with FAdV-8a at 5 dpc. **Necrosis in liver (A), lymphocytic infiltration and degeneration of glandular acini in pancreas (B), lymphocytic depletion and necrotic area in bursa of Fabricius (C), immunohistochemistry showing aggregation of viral material in the nuclei of hepatocytes (D); bar in lower right corner indicates magnification.

**Additional file 4. Individual distribution of B cells in PBMC for each experimental group.** Negative control (A), vaccination-only (B), challenge control (C) and vaccinated/challenged group (D). The asterisk indicates statistical significance (p ≤ 0.05) compared to the negative control.

**Additional file 5. Individual distribution of monocytes/macrophages in PBMC for each experimental group.** Negative control (A), vaccination-only (B), challenge control (C) and vaccinated/challenged group (D). The asterisk indicates statistical significance (p ≤ 0.05) compared to the negative control.

**Additional file 6. Individual distribution of TCRαβ**^**+**^** T cells in PBMC for each experimental group. **Negative control (A), vaccination-only (B), challenge control (C) and vaccinated/challenged group (D). The asterisk indicates statistical significance (p ≤ 0.05) compared to the negative control.

**Additional file 7. Individual distribution of TCR **${\varvec{\upgamma}}$**δ**^**+**^** T cells in PBMC for each experimental group.** Negative control (A), vaccination-only (B), challenge control (C) and vaccinated/challenged group (D). The asterisk indicates statistical significance (p ≤ 0.05) compared to the negative control.

**Additional file 8. Individual distribution of CD4**^**+**^** T cells in PBMC for each experimental group. **Negative control (A), vaccination-only (B), challenge control (C) and vaccinated/challenged group (D). The asterisk indicates statistical significance (p ≤ 0.05) compared to the negative control.

**Additional file 9. Individual distribution of CD8α**^**+**^** T cells in PBMC for each experimental group. **Negative control (A), vaccination-only (B), challenge control (C) and vaccinated/challenged group (D). The asterisk indicates statistical significance (p ≤ 0.05) compared to the negative control.

## Data Availability

The datasets supporting the conclusions of this article are included within the article and its additional files.

## Abbreviations

AGE: adenoviral gizzard erosion
AST: aspartate transaminase
CD: cluster of differentiation
CEL: chicken embryo liver
dpc: days post challenge
dpv: days post vaccination
ELISA: Eenzyme-linked immunosorbent assay
FAdV: Fowl adenovirus
FBS: fetal bovine serum
FCM: flow cytometry
HHS: hepatitis-hydropericardium syndrome
IBH: inclusion body hepatitis
IHC: immunohistochemistry
mAbs: monoclonal antibodies
nAbs: neutralizing antibodies
OD: optical density
PBMCs: peripheral blood mononuclear cells
PBS: phosphate buffered saline
qPCR: quantitative polymerase chain reaction
SPF: specific pathogen-free
TCID_50_: 50% tissue culture infective dose
TCR: T cell receptor
VNT: virus neutralization test
