# Supplementary figures and images for: Fowl adenovirus (FAdV) fiber-based vaccine against inclusion body hepatitis (IBH) provides type-specific protection guided by humoral immunity and regulation of B and T cell response
Source: Vet Res. 2020 Dec 2;51:143. doi: 10.1186/s13567-020-00869-8 (PMC7709361; doi:10.1186/s13567-020-00869-8)

## Slide 1
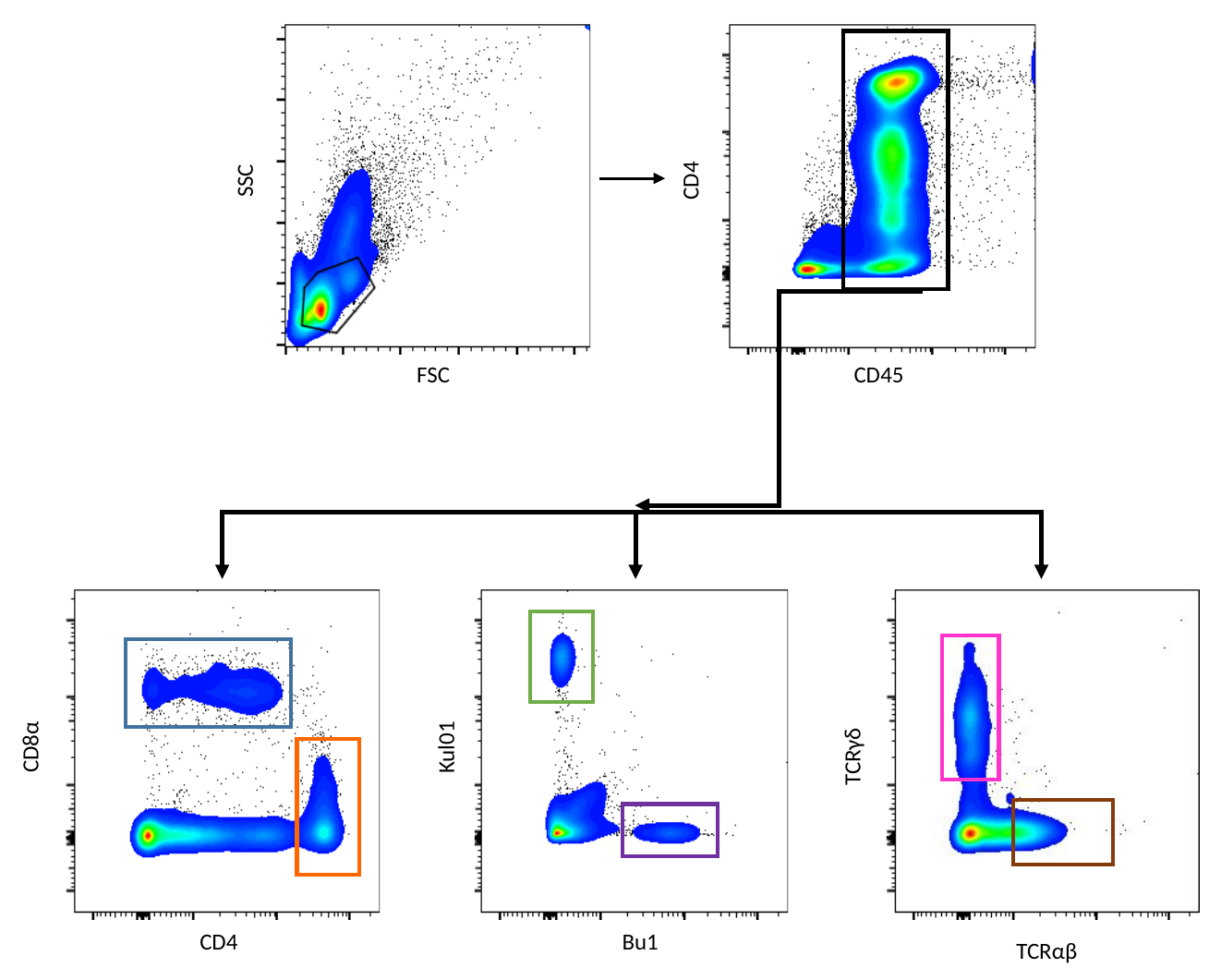

CD4
CD45
SSC
FSC
Kul01
Bu1
TCRγδ
TCRαβ
CD8α
CD4

Supplement: Supplementary file 1 — Additional file 1. Gating strategy for peripheral blood mononuclear cells in multicolor flow cytometry analysis applying three different panels of antibody combination. The cells were gated according to their light scatter properties. Potential leukocytes were gated with FSC/SSC and afterwards for CD45+ cells (double lined). In the first panel, CD45+ cells were further analyzed for CD45+CD4+CD8α− T cells (orange gate) and CD45+CD4−CD8α+ T cells (blue gate). In the second panel, B cells and monocytes/macrophages were identified by CD45+Bu1+Kul01− (purple gate) and CD45+Bu1−Kul01+ phenotype (green gate) respectively. The last panel analyzed CD45+TCRαβ+TCRδγ− T cells (brown gate) and CD45+TCRαβ−TCRδγ+ T cells (pink gate). The gating strategy is shown as a representative example for isolated PBMCs from a bird at 14 dpc and was performed accordingly for all analyzed samples. [file 13567_2020_869_MOESM1_ESM.pptx]

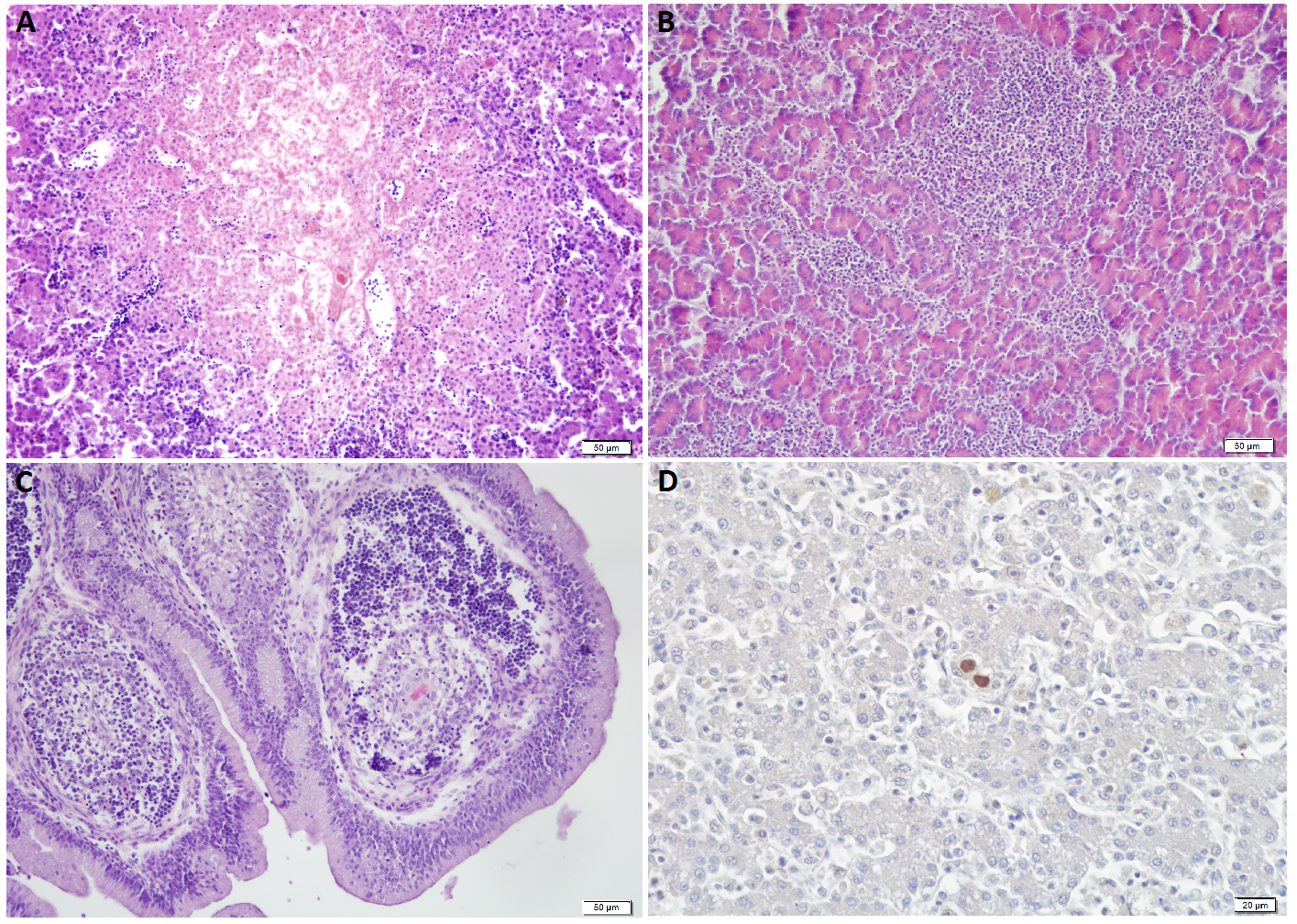

Supplement: Supplementary file 3 — Additional file 3. Histopathological lesions in different organs from a challenge control bird infected with FAdV-8a at 5 dpc. Necrosis in liver (A), lymphocytic infiltration and degeneration of glandular acini in pancreas (B), lymphocytic depletion and necrotic area in bursa of Fabricius (C), immunohistochemistry showing aggregation of viral material in the nuclei of hepatocytes (D); bar in lower right corner indicates magnification. [file 13567_2020_869_MOESM3_ESM.png]
