# Supplementary material for: Fowl adenovirus (FAdV) fiber-based vaccine against inclusion body hepatitis (IBH) provides type-specific protection guided by humoral immunity and regulation of B and T cell response
Source: Vet Res. 2020 Dec 2;51:143. doi: 10.1186/s13567-020-00869-8 (PMC7709361; doi:10.1186/s13567-020-00869-8)
Supplement: Supplementary file 2 — Additional file 2. Antibody panels. List of antibodies and antibody combinations used in this study. [file 13567_2020_869_MOESM2_ESM.docx]

| **species specificity** | **antigen** | **clone** | **isotype** | **fluorochrome** | **labeling strategy** | **source of primary mAb** |
| --- | --- | --- | --- | --- | --- | --- |
| **panel 1** | | | | | | |
| chicken | CD45 | LT40 | mouse IgM | APC | directly conjugated | Southern-Biotech |
| chicken | CD4 | CT4 | mouse IgG1 | BV421 | biotin-streptavidin^a^ | Southern-Biotech |
| chicken | CD8α | 3-298 | mouse IgG2b | R-PE | directly conjugated | Southern-Biotech |
| **panel 2** | | | | | | |
| chicken | CD45 | LT40 | mouse IgM | APC | directly conjugated | Southern-Biotech |
| chicken | Bu-1 | AV20 | mouse IgG1 | BV421 | biotin-streptavidin^a^ | Southern-Biotech |
| chicken | monocytes/ macrophags | Kul-01 | mouse IgG1 | R-PE | directly conjugated | Southern-Biotech |
| **panel 3** | | | | | | |
| chicken | CD45 | LT40 | mouse IgM | APC | directly conjugated | Southern-Biotech |
| chicken | TCR-γδ | TCR1 | mouse IgG1 | BV421 | biotin-streptavidin^a^ | Southern-Biotech |
| chicken | TCR-αβ/ VB1 | TCR2 | mouse IgG1 | FITC | directly conjugated | Southern-Biotech |

^a^Brilliant Violet 421™ Streptavidin, BioLegend
