# Supplementary material for: Fowl adenovirus (FAdV) fiber-based vaccine against inclusion body hepatitis (IBH) provides type-specific protection guided by humoral immunity and regulation of B and T cell response
Source: Vet Res. 2020 Dec 2;51:143. doi: 10.1186/s13567-020-00869-8 (PMC7709361; doi:10.1186/s13567-020-00869-8)
Supplement: Supplementary file 4 — Additional file 4. Individual distribution of B cells in PBMC for each experimental group. Negative control (A), vaccination-only (B), challenge control (C) and vaccinated/challenged group (D). The asterisk indicates statistical significance (p ≤ 0.05) compared to the negative control. [file 13567_2020_869_MOESM4_ESM.pptx]

## Slide 1
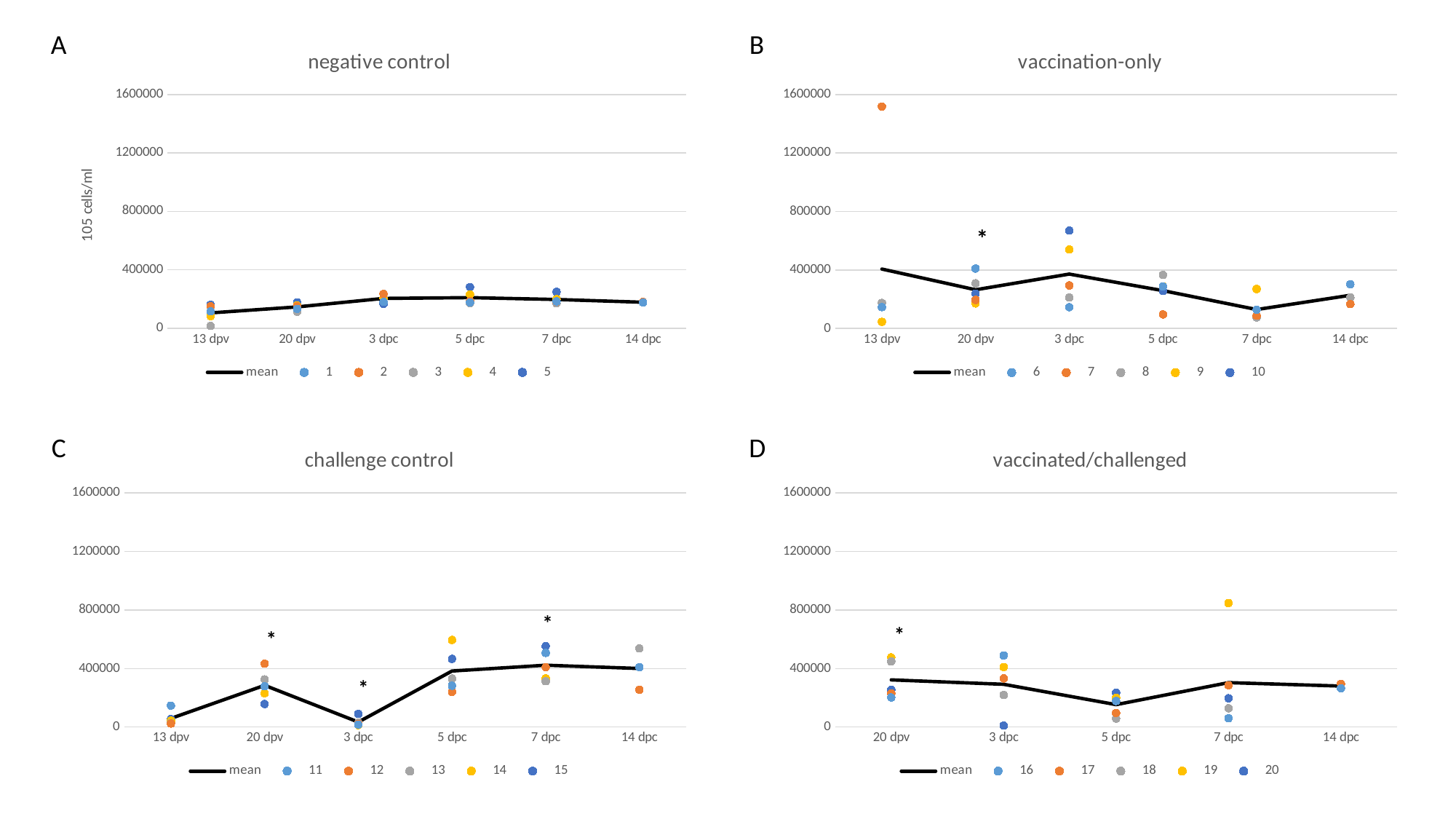

A
B
### Chart: negative control
| Category | mean | 1 | 2 | 3 | 4 | 5 |
|---|---|---|---|---|---|---|
| 13 dpv | 105695.28090000001 | 115892.7 | 150688.076 | 16615.368000000002 | 83533.1805 | 161747.08 |
| 20 dpv | 146510.29100000003 | 133066.73 | 150693.04799999998 | 114269.727 | 156791.39 | 177730.56 |
| 3 dpc | 204841.668 | 180622.22 | 234689.4 | 235343.68 | 206547.6 | 167005.44000000003 |
| 5 dpc | 209952.1504 | 177569.35199999998 | 186425.69400000002 | 171845.66199999998 | 231523.47400000002 | 282396.57000000007 |
| 7 dpc | 197339.35185454547 | 184373.33000000005 | 182316.25 | 171114.272 | 199620.18 | 249272.72727272726 |
| 14 dpc | 178548.17833333334 | 176762.25 | 180263.88 | 178618.405 | None | None |
### Chart: vaccination-only
| Category | mean | 6 | 7 | 8 | 9 | 10 |
|---|---|---|---|---|---|---|
| 13 dpv | 406516.7556 | 146212.43 | 1518036.8 | 174569.822 | 45374.292 | 148390.434 |
| 20 dpv | 264654.75800000003 | 409550.4 | 195375.375 | 308998.215 | 171226.72 | 238123.08 |
| 3 dpc | 372126.525 | 145434.175 | 294151.5 | 211688.1 | 539804.93 | 669553.92 |
| 5 dpc | 258886.188 | 287708.52 | 96144.18 | 366109.2 | 288532.86 | 255936.17999999996 |
| 7 dpc | 129404.2788 | 128181.6 | 86811.49 | 75344.984 | 269607.144 | 87076.17600000002 |
| 14 dpc | 227020.33566666665 | 302257.44000000006 | 166758.40999999997 | 212045.157 | None | None |C
D
### Chart: challenge control
| Category | mean | 11 | 12 | 13 | 14 | 15 |
|---|---|---|---|---|---|---|
| 13 dpv | 58923.1919 | 146634.88 | 23905.1835 | 26800.224 | 42841.655999999995 | 54434.016 |
| 20 dpv | 284878.97699999996 | 278164.425 | 432729.6 | 325743.6 | 230515.34000000003 | 157241.92 |
| 3 dpc | 34051.926 | 15983.849999999999 | 21695.04 | 29494.920000000006 | 13085.1 | 90000.72 |
| 5 dpc | 383002.9 | 282331.98 | 240007.1 | 331308.6 | 595261.05 | 466105.77 |
| 7 dpc | 422542.30999999994 | 506691.36 | 408927.75 | 312858.0 | 331468.2 | 552766.24 |
| 14 dpc | 400198.76333333337 | 408932.44 | 254878.65 | 536785.2 | None | None |
### Chart: vaccinated/challenged
| Category | mean | 16 | 17 | 18 | 19 | 20 |
|---|---|---|---|---|---|---|
| 20 dpv | 322211.60599999997 | 202198.92 | 230063.96 | 447926.64 | 475770.15 | 255098.36 |
| 3 dpc | 292041.42213333334 | 489102.6666666666 | 332365.68 | 219047.4 | 409990.42 | 9700.944 |
| 5 dpc | 153085.6946 | 177948.68 | 96547.07699999999 | 57751.344 | 199176.872 | 234004.5 |
| 7 dpc | 303553.9632 | 60051.816000000006 | 285584.25 | 127944.78 | 847717.5 | 196471.46999999997 |
| 14 dpc | 280127.57999999996 | 264568.08 | 295687.07999999996 | None | None | None |*
*
*
*
*
