# Supplementary material for: Fowl adenovirus (FAdV) fiber-based vaccine against inclusion body hepatitis (IBH) provides type-specific protection guided by humoral immunity and regulation of B and T cell response
Source: Vet Res. 2020 Dec 2;51:143. doi: 10.1186/s13567-020-00869-8 (PMC7709361; doi:10.1186/s13567-020-00869-8)
Supplement: Supplementary file 5 — Additional file 5. Individual distribution of monocytes/macrophages in PBMC for each experimental group. Negative control (A), vaccination-only (B), challenge control (C) and vaccinated/challenged group (D). The asterisk indicates statistical significance (p ≤ 0.05) compared to the negative control. [file 13567_2020_869_MOESM5_ESM.pptx]

## Slide 1
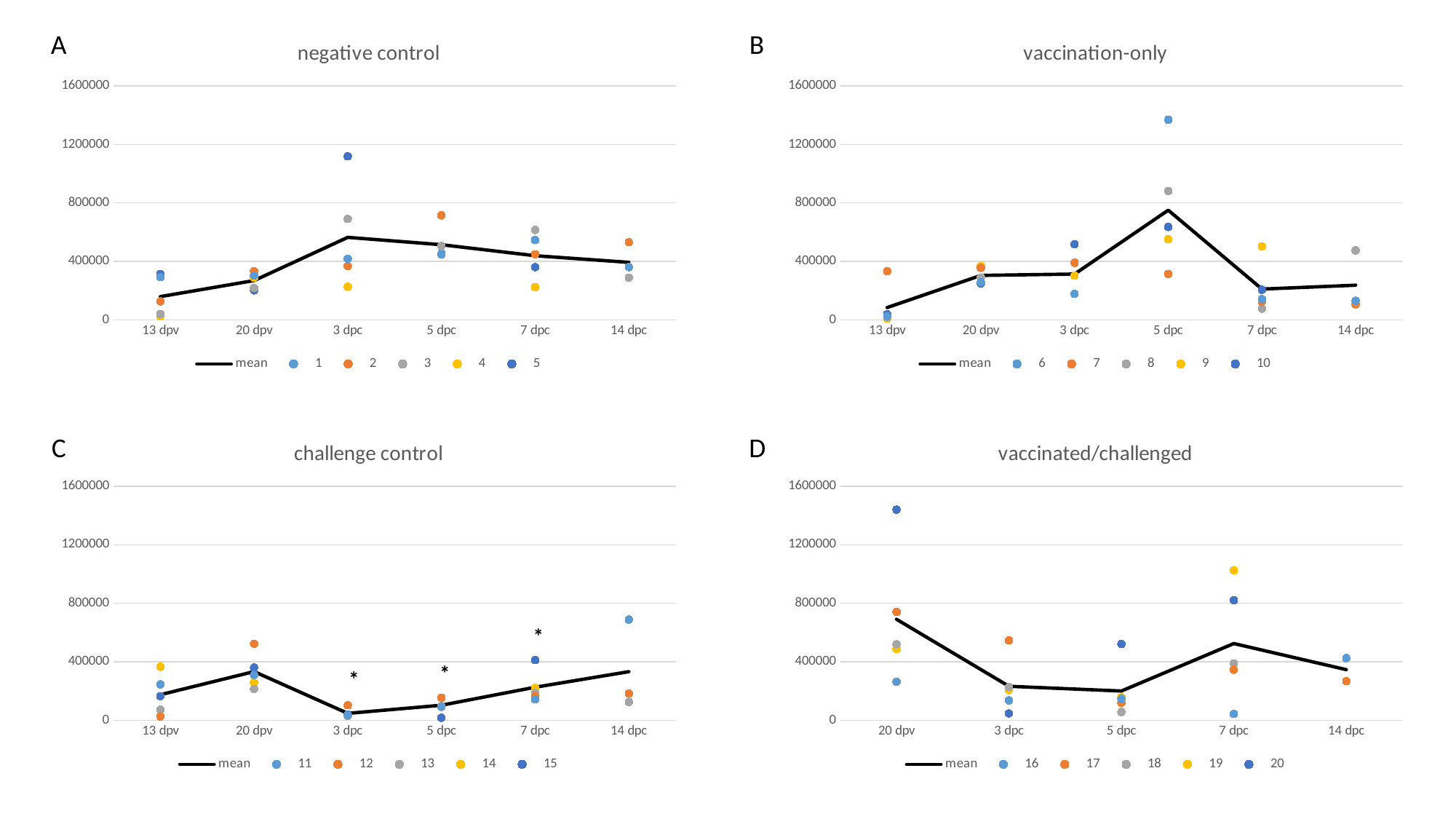

A
B
### Chart: negative control
| Category | mean | 1 | 2 | 3 | 4 | 5 |
|---|---|---|---|---|---|---|
| 13 dpv | 159321.4557 | 292076.4 | 126708.78000000001 | 39966.696 | 24568.5825 | 313286.82 |
| 20 dpv | 268897.79720000003 | 304013.77 | 332563.968 | 217699.638 | 288477.21 | 201734.4 |
| 3 dpc | 564352.642 | 418192.21 | 368339.4 | 689946.4000000001 | 226808.4 | 1118476.8 |
| 5 dpc | 514104.94230000005 | 447405.13200000004 | 714767.5079999999 | 504972.7035 | 450325.87799999997 | 453053.49000000005 |
| 7 dpc | 438739.75159999996 | 546740.29 | 448242.5 | 614614.528 | 223653.07636363633 | 360448.3636363636 |
| 14 dpc | 393586.125 | 360923.85 | 530776.98 | 289057.545 | None | None |
### Chart: vaccination-only
| Category | mean | 6 | 7 | 8 | 9 | 10 |
|---|---|---|---|---|---|---|
| 13 dpv | 84352.8154 | 24123.58 | 332929.60000000003 | 17817.575999999997 | 8674.497 | 38218.824 |
| 20 dpv | 304188.448 | 257976.6 | 355957.875 | 289564.36500000005 | 368995.2 | 248448.2 |
| 3 dpc | 313160.725 | 177590.475 | 390379.5 | 177921.90000000002 | 303063.11 | 516848.64 |
| 5 dpc | 750029.725 | 1368935.7000000002 | 314193.66 | 881125.2 | 550690.14 | 635203.925 |
| 7 dpc | 210683.7342 | 141854.30399999997 | 125340.185 | 77312.95 | 502804.32 | 206106.91200000004 |
| 14 dpc | 237091.18000000002 | 130705.92 | 105969.63 | 474597.99 | None | None |C
### Chart: challenge control
| Category | mean | 11 | 12 | 13 | 14 | 15 |
|---|---|---|---|---|---|---|
| 13 dpv | 174896.90118 | 245252.0 | 26223.261899999998 | 72304.771 | 365939.145 | 164765.32799999998 |
| 20 dpv | 332948.46400000004 | 309792.45 | 523187.2 | 214239.06 | 257388.89 | 360134.72 |
| 3 dpc | 47041.246199999994 | 35697.26499999999 | 102448.8 | 30197.180000000004 | 28351.05 | 38511.936 |
| 5 dpc | 102856.43599999999 | 92160.18 | 154132.99999999997 | 96913.4 | 153907.8 | 17167.8 |
| 7 dpc | 225765.123 | 143260.95 | 167252.24999999997 | 186339.6 | 220125.6 | 411847.215 |
| 14 dpc | 332284.445 | 688728.32 | 182753.415 | 125371.6 | None | None |D
### Chart: vaccinated/challenged
| Category | mean | 16 | 17 | 18 | 19 | 20 |
|---|---|---|---|---|---|---|
| 20 dpv | 690299.939 | 263515.56 | 740766.285 | 518939.4 | 487615.05000000005 | 1440663.4 |
| 3 dpc | 232376.39440000002 | 135792.0 | 545836.32 | 226983.9 | 206528.84 | 46740.912000000004 |
| 5 dpc | 200119.065 | 146249.31999999998 | 119443.617 | 56495.88 | 157428.008 | 520978.5 |
| 7 dpc | 524708.7736 | 42868.848 | 345633.75 | 388793.44 | 1025173.03 | 821074.8 |
| 14 dpc | 346052.7100000001 | 425104.4000000001 | 267001.02 | None | None | None |*
*
*
