# Supplementary material for: Fowl adenovirus (FAdV) fiber-based vaccine against inclusion body hepatitis (IBH) provides type-specific protection guided by humoral immunity and regulation of B and T cell response
Source: Vet Res. 2020 Dec 2;51:143. doi: 10.1186/s13567-020-00869-8 (PMC7709361; doi:10.1186/s13567-020-00869-8)
Supplement: Supplementary file 6 — Additional file 6. Individual distribution of TCRαβ+ T cells in PBMC for each experimental group. Negative control (A), vaccination-only (B), challenge control (C) and vaccinated/challenged group (D). The asterisk indicates statistical significance (p ≤ 0.05) compared to the negative control. [file 13567_2020_869_MOESM6_ESM.pptx]

## Slide 1
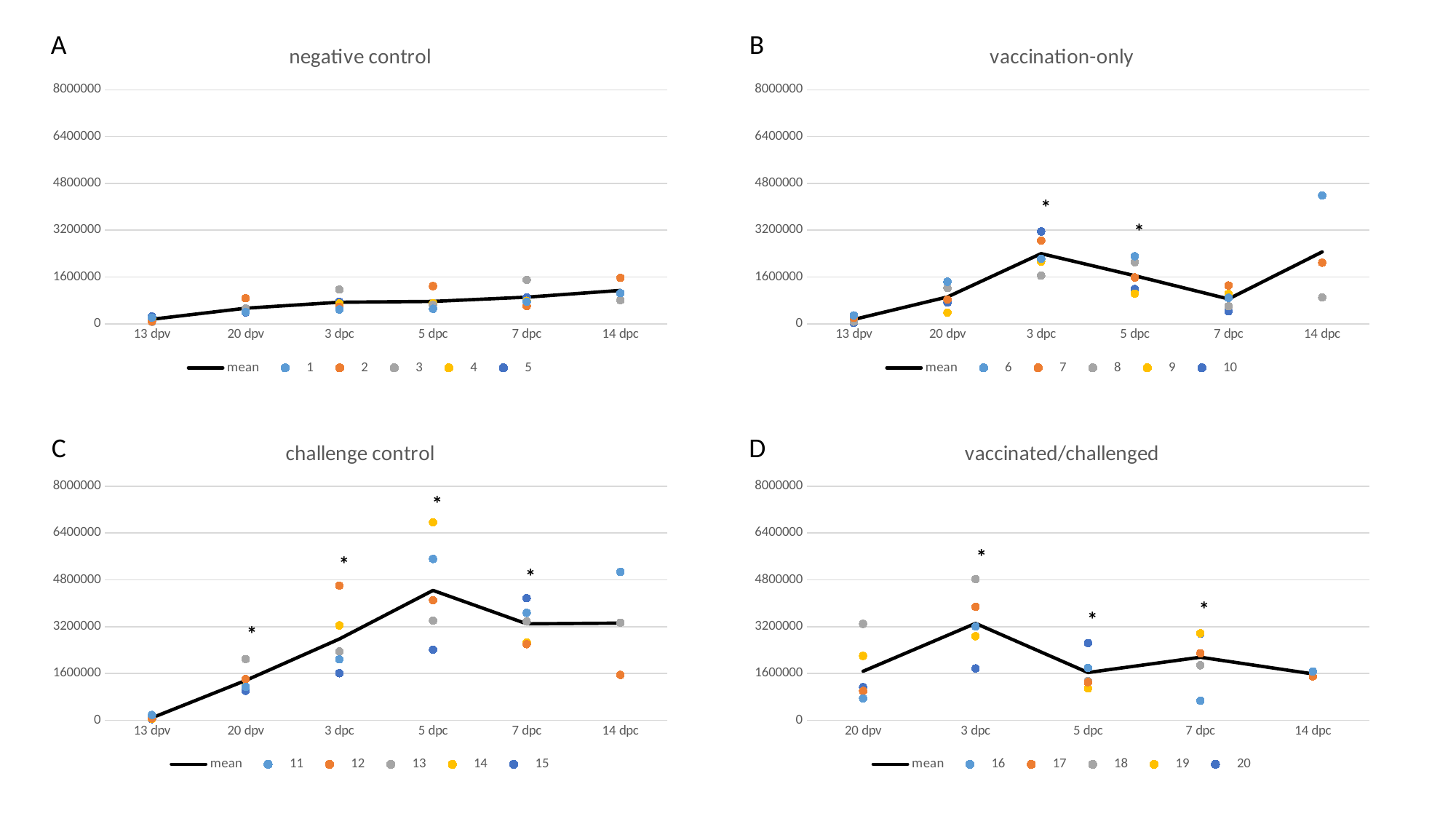

A
B
### Chart: negative control
| Category | mean | 1 | 2 | 3 | 4 | 5 |
|---|---|---|---|---|---|---|
| 13 dpv | 153644.38760000002 | 210348.6 | 77932.712 | 68033.196 | 163790.55 | 248116.88 |
| 20 dpv | 531878.851 | 403570.995 | 873925.2 | 523924.44 | 470847.86 | 387125.76 |
| 3 dpc | 735513.74 | 488945.5 | 577368.0000000001 | 1168252.8 | 692244.0 | 750758.4 |
| 5 dpc | 762853.9860000001 | 510656.9600000001 | 1286255.88 | 640899.805 | 706019.385 | 670437.9000000001 |
| 7 dpc | 909076.9098181818 | 752804.6000000001 | 603850.0 | 1501615.04 | 784747.6363636362 | 902367.2727272728 |
| 14 dpc | 1139616.2833333332 | 1044130.5 | 1568963.4 | 805754.95 | None | None |
### Chart: vaccination-only
| Category | mean | 6 | 7 | 8 | 9 | 10 |
|---|---|---|---|---|---|---|
| 13 dpv | 146071.874 | 286835.25 | 201590.4 | 71270.30399999999 | 141609.31000000003 | 29054.106000000007 |
| 20 dpv | 916819.43 | 1435434.0 | 826107.75 | 1217854.6 | 381942.4 | 722758.4 |
| 3 dpc | 2397282.8400000003 | 2221708.0 | 2843100.0 | 1642855.5000000002 | 2123960.3 | 3154790.4 |
| 5 dpc | 1642788.2900000003 | 2308628.85 | 1582468.8 | 2104848.0 | 1031045.4 | 1186950.4 |
| 7 dpc | 846999.8759999999 | 884453.04 | 1307049.4 | 598823.94 | 1018612.2 | 426060.80000000005 |
| 14 dpc | 2455858.2866666666 | 4384094.4 | 2086533.7999999998 | 896946.66 | None | None |
### Chart: challenge control
| Category | mean | 11 | 12 | 13 | 14 | 15 |
|---|---|---|---|---|---|---|
| 13 dpv | 82631.77192 | 181228.32 | 64326.6756 | 79283.996 | 49981.932 | 38337.936 |
| 20 dpv | 1360442.25 | 1143474.75 | 1405760.0 | 2092276.2 | 1152576.7000000002 | 1008123.6 |
| 3 dpc | 2777924.154 | 2083228.4499999997 | 4604169.600000001 | 2352571.0000000005 | 3242197.0 | 1607454.72 |
| 5 dpc | 4440851.22 | 5514982.2 | 4106543.4999999995 | 3403238.0 | 6767416.5 | 2412075.9 |
| 7 dpc | 3296787.66 | 3672004.35 | 2600812.5 | 3376116.0 | 2661984.0 | 4173021.45 |
| 14 dpc | 3319771.233333333 | 5073222.0 | 1550963.7 | 3335128.0 | None | None |C
D
### Chart: vaccinated/challenged
| Category | mean | 16 | 17 | 18 | 19 | 20 |
|---|---|---|---|---|---|---|
| 20 dpv | 1674340.2 | 744559.2 | 1001571.55 | 3295720.4 | 2201177.25 | 1128672.6 |
| 3 dpc | 3310963.768 | 3206200.0 | 3877599.6 | 4825392.000000001 | 2873000.2 | 1772627.04 |
| 5 dpc | 1631223.898 | 1786691.2 | 1301286.69 | 1339161.6 | 1087210.0 | 2641770.0 |
| 7 dpc | 2154397.716 | 667832.8800000001 | 2290259.9999999995 | 1879498.9 | 2972662.7 | 2961734.1 |
| 14 dpc | 1584617.2000000002 | 1668732.8 | 1500501.6 | None | None | None |*
*
*
*
*
*
*
*
*
