# Supplementary material for: Fowl adenovirus (FAdV) fiber-based vaccine against inclusion body hepatitis (IBH) provides type-specific protection guided by humoral immunity and regulation of B and T cell response
Source: Vet Res. 2020 Dec 2;51:143. doi: 10.1186/s13567-020-00869-8 (PMC7709361; doi:10.1186/s13567-020-00869-8)
Supplement: Supplementary file 7 — Additional file 7. Individual distribution of TCR ${\varvec{\upgamma}}$γδ+ T cells in PBMC for each experimental group. Negative control (A), vaccination-only (B), challenge control (C) and vaccinated/challenged group (D). The asterisk indicates statistical significance (p ≤ 0.05) compared to the negative control. [file 13567_2020_869_MOESM7_ESM.pptx]

## Slide 1
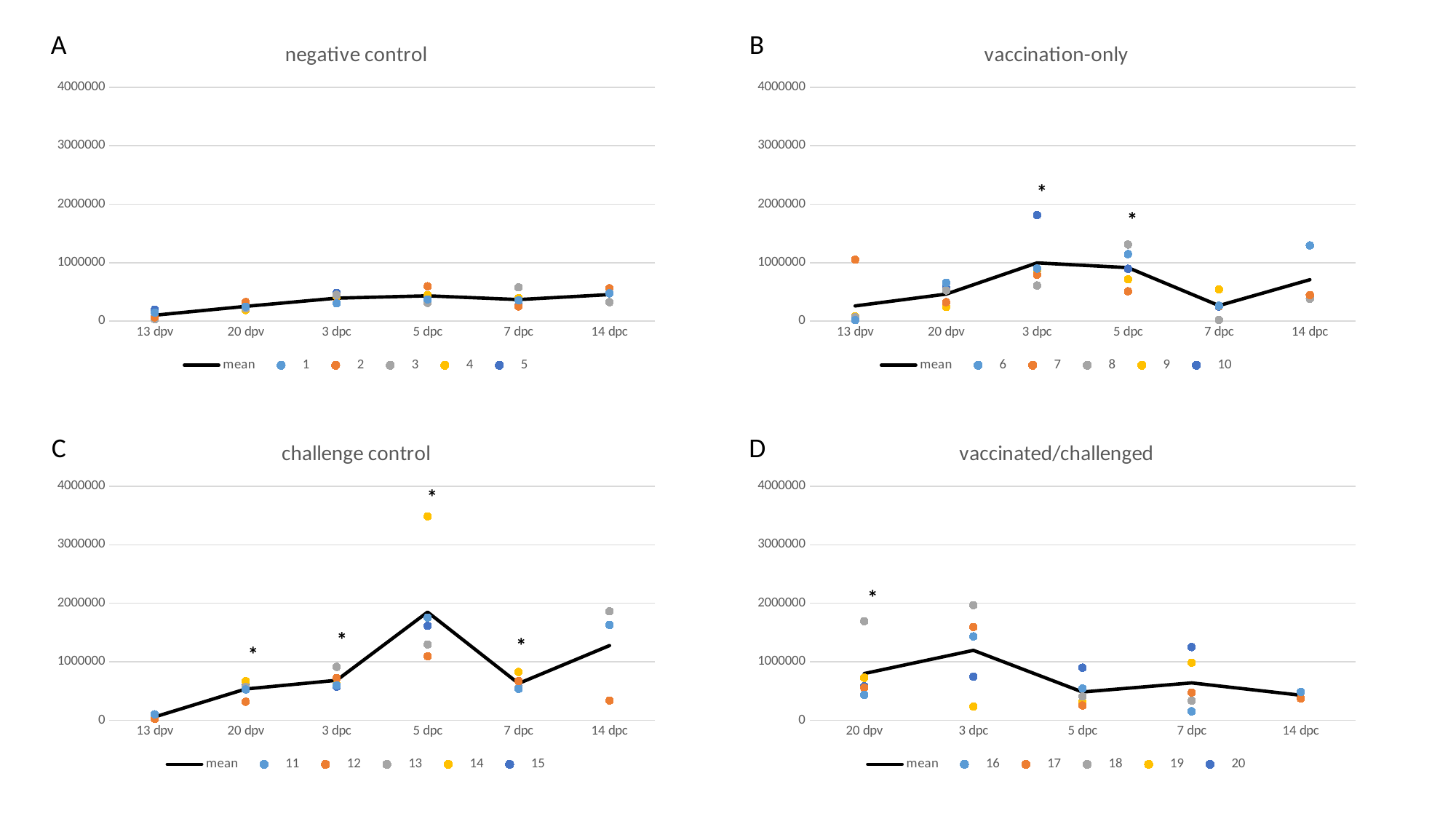

A
B
### Chart: negative control
| Category | mean | 1 | 2 | 3 | 4 | 5 |
|---|---|---|---|---|---|---|
| 13 dpv | 102093.8336 | 145368.3 | 65943.064 | 33904.332 | 67388.11200000001 | 197865.36 |
| 20 dpv | 253072.898 | 246707.66 | 325950.48 | 221312.91 | 189476.0 | 281917.43999999994 |
| 3 dpc | 393449.94000000006 | 304871.9 | 309533.4 | 448676.80000000005 | 421537.2 | 482630.4 |
| 5 dpc | 433247.7260000001 | 371386.88 | 596725.0380000001 | 312702.76200000005 | 445237.45 | 440186.50000000006 |
| 7 dpc | 369115.1648969697 | 356625.2300000001 | 251991.25 | 577365.1626666668 | 392373.8181818181 | 267220.36363636365 |
| 14 dpc | 454633.33499999996 | 476847.0 | 561933.7 | 325119.30499999993 | None | None |
### Chart: vaccination-only
| Category | mean | 6 | 7 | 8 | 9 | 10 |
|---|---|---|---|---|---|---|
| 13 dpv | 260233.00200000004 | 19122.35 | 1050713.6 | 67876.48 | 81555.1 | 81897.48000000001 |
| 20 dpv | 462612.249 | 657489.0 | 324733.5 | 525361.7450000001 | 242112.64 | 563364.36 |
| 3 dpc | 995911.4199999999 | 898914.75 | 793881.0 | 609739.6500000001 | 864695.3 | 1812326.4 |
| 5 dpc | 915412.5780000001 | 1146193.6200000003 | 510622.2 | 1309931.9999999998 | 714538.44 | 895776.63 |
| 7 dpc | 266562.3554 | 263199.55199999997 | 255069.71500000003 | 21085.35 | 541815.0 | 251642.16 |
| 14 dpc | 708572.5673333333 | 1294805.52 | 446879.68 | 384032.50200000004 | None | None |
### Chart: challenge control
| Category | mean | 11 | 12 | 13 | 14 | 15 |
|---|---|---|---|---|---|---|
| 13 dpv | 57479.50938 | 102231.36 | 24050.0634 | 46900.392 | 40759.075500000006 | 73456.656 |
| 20 dpv | 534559.883 | 526322.775 | 316601.6 | 566292.72 | 668852.8 | 594729.52 |
| 3 dpc | 683248.082 | 596730.3999999999 | 723168.0 | 912938.0000000001 | 609911.05 | 573492.96 |
| 5 dpc | 1849008.7 | 1755432.0 | 1094344.3 | 1295935.0 | 3485559.0 | 1613773.2 |
| 7 dpc | 628388.753 | 536851.56 | 670609.5000000001 | 553518.0000000001 | 825471.0 | 555493.705 |
| 14 dpc | 1276582.0616666665 | 1629580.4 | 337849.785 | 1862316.0 | None | None |
### Chart: vaccinated/challenged
| Category | mean | 16 | 17 | 18 | 19 | 20 |
|---|---|---|---|---|---|---|
| 20 dpv | 798404.4880000001 | 435056.16 | 555326.8 | 1693381.2 | 726487.2 | 581771.08 |
| 3 dpc | 1195045.892 | 1433360.0 | 1594274.4 | 1968252.0000000002 | 234134.18 | 745208.88 |
| 5 dpc | 483276.91 | 543211.76 | 252625.158 | 406351.84800000006 | 315725.78400000004 | 898470.0 |
| 7 dpc | 639101.28 | 152343.84 | 473413.5 | 335235.16 | 983352.2999999998 | 1251161.6 |
| 14 dpc | 427848.53 | 484249.36 | 371447.7 | None | None | None |C
D
*
*
*
*
*
*
*
