# Supplementary material for: Fowl adenovirus (FAdV) fiber-based vaccine against inclusion body hepatitis (IBH) provides type-specific protection guided by humoral immunity and regulation of B and T cell response
Source: Vet Res. 2020 Dec 2;51:143. doi: 10.1186/s13567-020-00869-8 (PMC7709361; doi:10.1186/s13567-020-00869-8)
Supplement: Supplementary file 8 — Additional file 8. Individual distribution of CD4+ T cells in PBMC for each experimental group. Negative control (A), vaccination-only (B), challenge control (C) and vaccinated/challenged group (D). The asterisk indicates statistical significance (p ≤ 0.05) compared to the negative control. [file 13567_2020_869_MOESM8_ESM.pptx]

## Slide 1
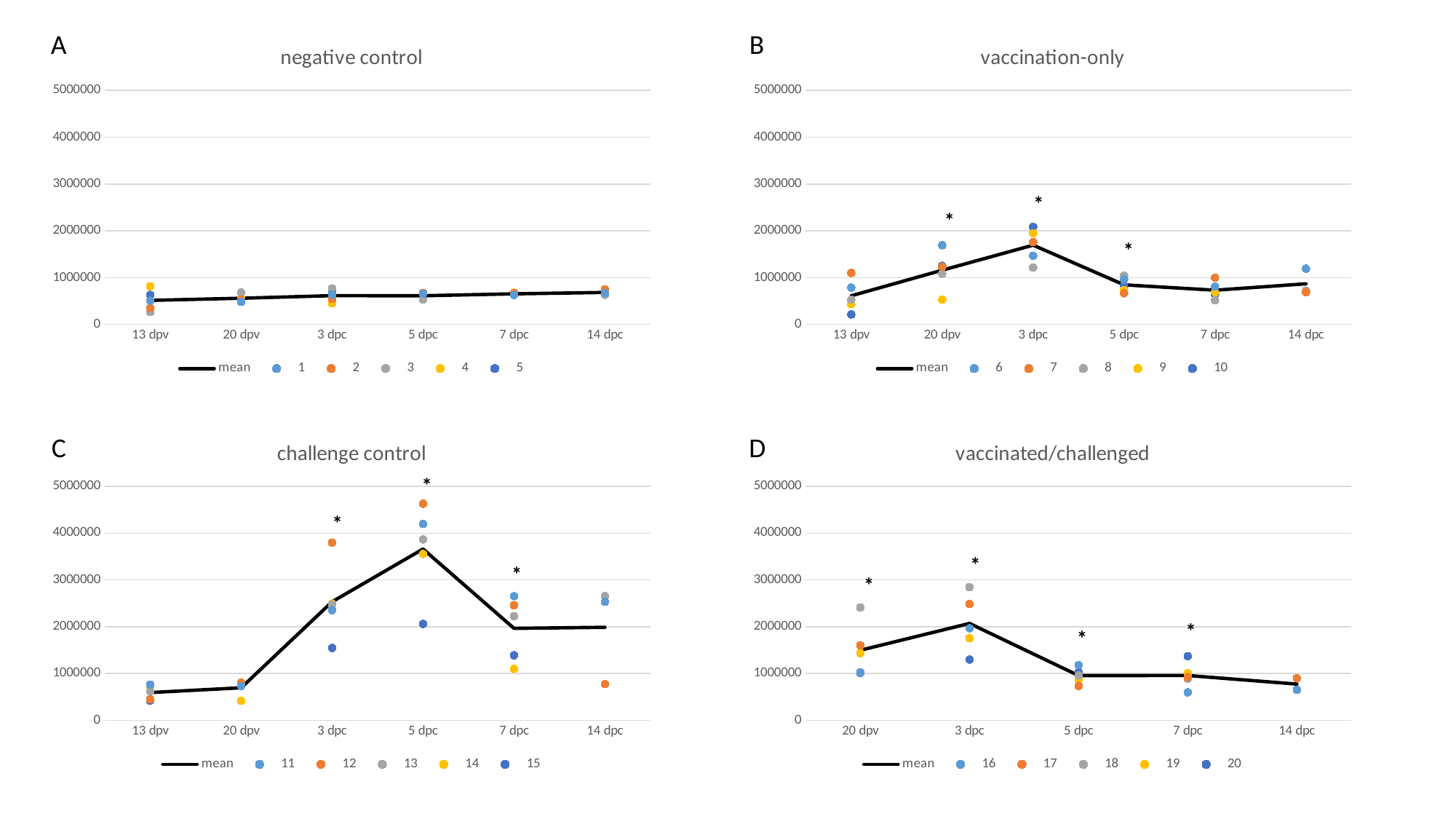

A
B
### Chart: negative control
| Category | mean | 1 | 2 | 3 | 4 | 5 |
|---|---|---|---|---|---|---|
| 13 dpv | 513684.2640000001 | 506929.5 | 353834.24 | 265816.32 | 812004.06 | 629837.2 |
| 20 dpv | 560026.4680000001 | 483847.65 | 528719.4 | 681817.29 | 559378.4 | 546369.6 |
| 3 dpc | 615276.4 | 634522.0 | 544544.0 | 769841.6000000001 | 451308.0 | 676166.3999999999 |
| 5 dpc | 613096.1309999999 | 654843.7399999999 | 673689.2400000001 | 532135.45 | 659559.8250000001 | 545252.4 |
| 7 dpc | 654036.9234545454 | 630712.6000000001 | 673443.75 | 649872.14 | 677008.8545454545 | 639147.2727272727 |
| 14 dpc | 684001.85 | 667585.7999999999 | 750834.0 | 633585.7499999999 | None | None |
### Chart: vaccination-only
| Category | mean | 6 | 7 | 8 | 9 | 10 |
|---|---|---|---|---|---|---|
| 13 dpv | 612447.505 | 786006.55 | 1100822.36 | 527152.34 | 434166.525 | 214089.75 |
| 20 dpv | 1158594.06 | 1692222.0 | 1233035.7 | 1084915.8 | 530216.4 | 1252580.4000000001 |
| 3 dpc | 1694805.9 | 1468958.25 | 1757193.75 | 1214513.9999999998 | 1950115.5 | 2083248.0 |
| 5 dpc | 846771.32 | 960845.3999999999 | 666019.2 | 1041000.0000000001 | 736288.0 | 829703.9999999999 |
| 7 dpc | 731533.324 | 806846.4 | 999846.75 | 515363.4200000001 | 687858.37 | 647751.68 |
| 14 dpc | 868697.5466666667 | 1192725.0 | 689474.1 | 723893.54 | None | None |
### Chart: challenge control
| Category | mean | 11 | 12 | 13 | 14 | 15 |
|---|---|---|---|---|---|---|
| 13 dpv | 591951.6451999999 | 760972.8 | 447678.891 | 612556.2100000001 | 719228.565 | 419321.76 |
| 20 dpv | 694336.02 | 733837.5 | 803635.2 | 781042.5 | 414403.5 | 738761.4 |
| 3 dpc | 2532255.1859999998 | 2352066.7499999995 | 3795838.4 | 2473532.0 | 2494996.25 | 1544842.53 |
| 5 dpc | 3660551.5200000005 | 4195978.199999999 | 4628757.0 | 3864759.6000000006 | 3554605.5 | 2058657.3 |
| 7 dpc | 1963497.4100000001 | 2651200.15 | 2456338.5 | 2223244.8 | 1098090.0000000002 | 1388613.6 |
| 14 dpc | 1986553.3999999997 | 2531634.0 | 772925.4 | 2655100.8 | None | None |
### Chart: vaccinated/challenged
| Category | mean | 16 | 17 | 18 | 19 | 20 |
|---|---|---|---|---|---|---|
| 20 dpv | 1496043.5 | 1023907.5 | 1598432.15 | 2409467.2 | 1435420.25 | 1012990.4 |
| 3 dpc | 2068996.4433333334 | 1966346.6666666665 | 2483109.9 | 2845111.5 | 1752458.4 | 1297955.75 |
| 5 dpc | 954952.782 | 1177272.0 | 735676.8 | 957646.4 | 874690.4600000002 | 1029478.25 |
| 7 dpc | 956402.04 | 596497.65 | 921472.4999999999 | 890820.0 | 1002403.35 | 1370816.7 |
| 14 dpc | 773340.3 | 649740.0000000001 | 896940.6 | None | None | None |C
D
*
*
*
*
*
*
*
*
*
*
