# Supplementary material for: Fowl adenovirus (FAdV) fiber-based vaccine against inclusion body hepatitis (IBH) provides type-specific protection guided by humoral immunity and regulation of B and T cell response
Source: Vet Res. 2020 Dec 2;51:143. doi: 10.1186/s13567-020-00869-8 (PMC7709361; doi:10.1186/s13567-020-00869-8)
Supplement: Supplementary file 9 — Additional file 9. Individual distribution of CD8α+ T cells in PBMC for each experimental group. Negative control (A), vaccination-only (B), challenge control (C) and vaccinated/challenged group (D). The asterisk indicates statistical significance (p ≤ 0.05) compared to the negative control. [file 13567_2020_869_MOESM9_ESM.pptx]

## Slide 1
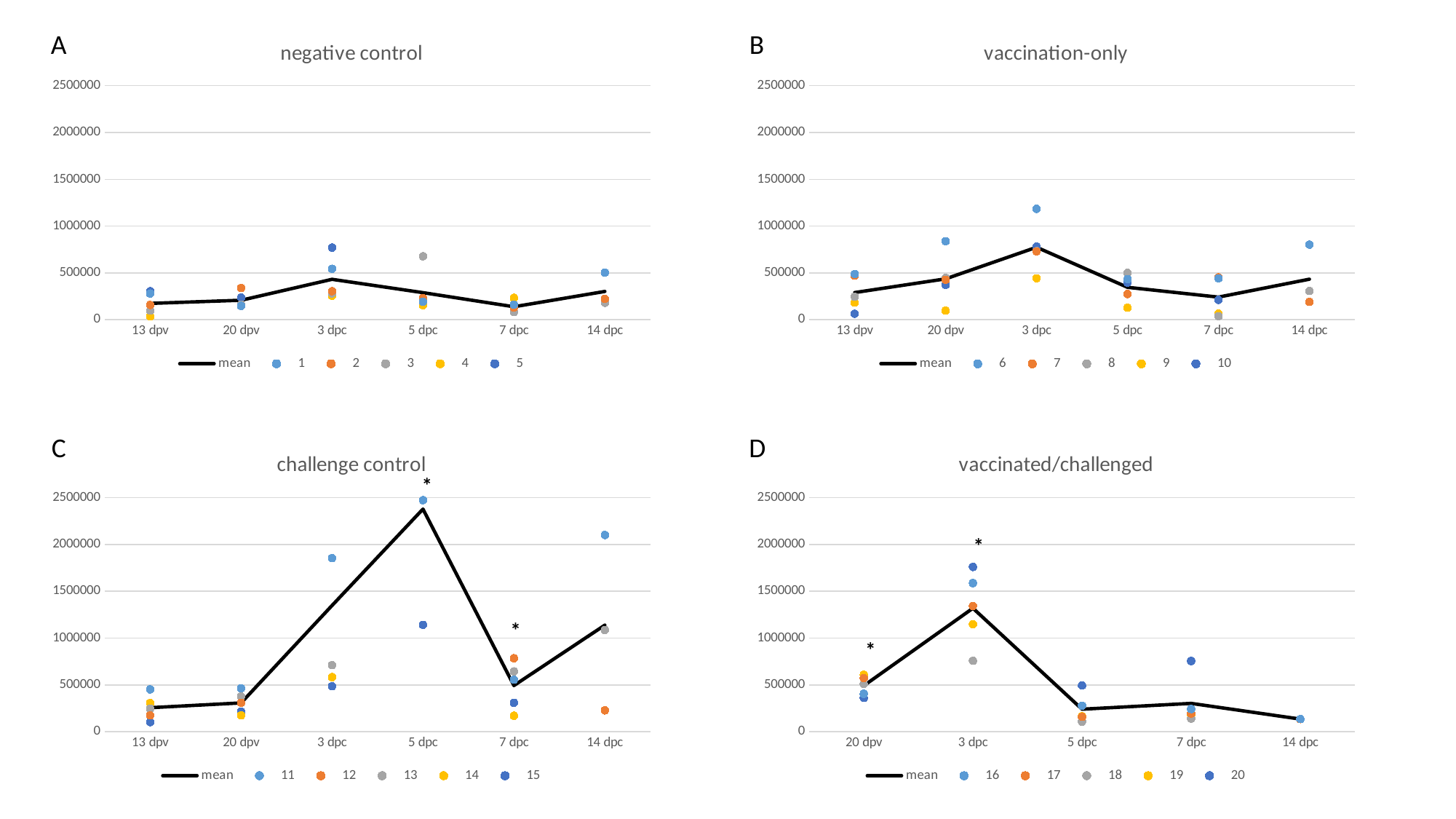

A
B
### Chart: negative control
| Category | mean | 1 | 2 | 3 | 4 | 5 |
|---|---|---|---|---|---|---|
| 13 dpv | 173898.818 | 279382.95 | 157013.944 | 95785.536 | 33750.780000000006 | 303560.88 |
| 20 dpv | 207191.7284 | 147346.65 | 338380.416 | 162042.29100000003 | 152330.725 | 235858.56 |
| 3 dpc | 430934.27999999997 | 543876.0 | 303875.0 | 280574.8 | 256267.19999999995 | 770078.3999999999 |
| 5 dpc | 288716.521 | 192139.78 | 230766.48000000004 | 677263.3 | 154433.52000000002 | 188979.525 |
| 7 dpc | 137963.6027090909 | 160760.58000000005 | 128733.125 | 80837.754 | 233040.73636363633 | 86445.81818181818 |
| 14 dpc | 301585.155 | 502607.7 | 222032.34 | 180115.425 | None | None |
### Chart: vaccination-only
| Category | mean | 6 | 7 | 8 | 9 | 10 |
|---|---|---|---|---|---|---|
| 13 dpv | 289655.206 | 487523.04999999993 | 469537.64 | 245435.3 | 182572.59 | 63207.45 |
| 20 dpv | 435924.62399999995 | 838240.2 | 426495.225 | 445950.855 | 97318.2 | 371618.64 |
| 3 dpc | 773871.399 | 1183936.5 | 729911.25 | 732564.0 | 442748.445 | 780196.8 |
| 5 dpc | 345813.81599999993 | 432998.99999999994 | 273543.6 | 499680.00000000006 | 127699.95000000001 | 395146.5299999999 |
| 7 dpc | 240884.17080000002 | 441076.032 | 451093.65 | 36791.612 | 64143.848 | 211315.712 |
| 14 dpc | 432513.2863333333 | 801511.2 | 190426.18 | 305602.479 | None | None |
### Chart: challenge control
| Category | mean | 11 | 12 | 13 | 14 | 15 |
|---|---|---|---|---|---|---|
| 13 dpv | 255910.36299999998 | 451476.48 | 173855.88 | 246183.728 | 305878.815 | 102156.912 |
| 20 dpv | 307048.659 | 463296.075 | 308736.0 | 376057.5 | 174049.47 | 213104.25 |
| 3 dpc | 1348146.518 | 1854618.1499999994 | 3108533.68 | 710421.4 | 582414.05 | 484745.31 |
| 5 dpc | 2378567.249 | 2473603.92 | 2821337.6 | 2915721.0000000005 | 2541231.125 | 1140942.6 |
| 7 dpc | 492570.1249999999 | 554864.18 | 784262.325 | 644476.32 | 170667.0 | 308580.8 |
| 14 dpc | 1138174.9133333333 | 2101084.0 | 227263.14 | 1086177.6 | None | None |
### Chart: vaccinated/challenged
| Category | mean | 16 | 17 | 18 | 19 | 20 |
|---|---|---|---|---|---|---|
| 20 dpv | 490594.02600000007 | 404253.85 | 572372.7100000001 | 509123.7200000001 | 607466.25 | 359753.6 |
| 3 dpc | 1319222.0266666666 | 1587733.3333333335 | 1343518.4 | 756756.0 | 1148162.4 | 1759940.0 |
| 5 dpc | 239880.6892 | 277967.0 | 157965.12000000002 | 107006.576 | 163735.16000000003 | 492729.59 |
| 7 dpc | 302113.362 | 242160.24 | 194109.0 | 140781.37499999997 | 179061.795 | 754454.4 |
| 14 dpc | 134321.61000000002 | 134546.16000000003 | 134097.06 | None | None | None |C
D
*
*
*
*
